# Supplementary material for: Studies on the selectivity of the SARS-CoV-2 papain-like protease reveal the importance of the P2′ proline of the viral polyprotein
Source: RSC Chem Biol. 2023 Oct 24;5(2):117–30. doi: 10.1039/d3cb00128h (PMC10849127; doi:10.1039/d3cb00128h)
Supplement: CB-005-D3CB00128H-s001 [file CB-005-D3CB00128H-s001.pdf]

Electronic Supplementary Material (ESI) for RSC Chemical Biology.
This journal is © The Royal Society of Chemistry 2023

Supplementary Information

Studies on the selectivity of the SARS-CoV-2 papain-like protease reveal the importance of the P2′ proline of the viral polyprotein

H. T. Henry Chan[a], Lennart Brewitz[a], Petra Lukacik[bc], Claire Strain-Damerell[bc], Martin A. Walsh[bc], Christopher J. Schofield[a]*, and Fernanda Duarte[a]*

[a]Chemistry Research Laboratory, Department of Chemistry and the Ineos Oxford Institute for Antimicrobial Research, University of Oxford, 12 Mansfield Road, Oxford, OX1 3TA, United Kingdom.

[b]Diamond Light Source Ltd., Harwell Science and Innovation Campus, Didcot, OX11 0DE, United Kingdom.

[c]Research Complex at Harwell, Harwell Science and Innovation Campus, Didcot, OX11 0FA, United Kingdom.

E-mail: christopher.schofield@chem.ox.ac.uk; fernanda.duartegonzalez@chem.ox.ac.uk

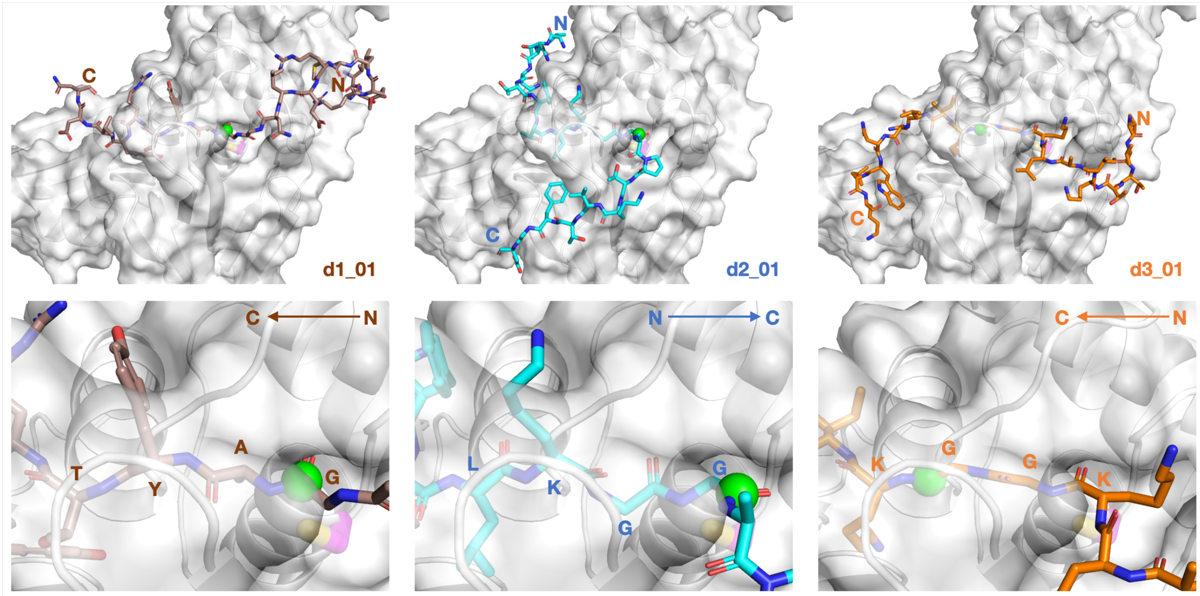

Figure S1: Top-ranked binding poses from AutoDock CrankPep (ADCP)[1] for the non-structural protein (nsp) peptides **1**, **2**, and **3**,[2] with the N- and C-terminals labeled. The P1 scissile amide carbonyl carbon is shown as a green sphere. The bottom row shows magnified views around the S4-S1 subsites, with each residue labeled and the N-/C-terminal peptide direction indicated.

Table S1: Deviations of P4-P1 Cα atom positions (compared to those in VIR251 in PDB 6WX4)[3] and root mean square deviation (RMSD) values for the 100 top-ranked solutions from ADCP[1] docking of peptides **1**, **2**, and **3**. Solutions that pass the filter (all four deviations within 2 Å) are in green, while reverse-bound solutions for peptide **3** that place Gly-Gly in S2-S1 are in orange.

Peptide 1					
**Ranking**	**Deviation (Å)**				**RMSD (Å)**
	P4	*P3*	*P2*	*P1*	
1	17.719	13.064	7.576	1.417	11.662
2	13.666	8.103	7.947	9.937	10.177
3	18.191	12.249	5.673	0.914	11.335
4	17.483	13.424	7.936	1.724	11.745
5	6.922	0.564	0.830	0.933	3.528
6	5.533	0.709	0.507	0.679	2.821
7	31.863	29.764	30.688	30.875	30.807
8	32.268	25.838	25.227	19.290	26.064
9	17.040	10.391	4.744	2.624	10.340
10	25.450	22.260	19.441	14.266	20.765
11	17.323	11.897	5.589	0.513	10.876
12	17.102	17.723	14.568	10.983	15.325
13	16.175	9.664	4.350	3.021	9.786
14	28.948	26.041	23.593	21.042	25.078
15	27.717	25.926	25.155	22.993	25.504
16	1.399	1.056	0.932	0.820	1.074
17	18.706	20.327	15.567	15.243	17.591
18	21.105	19.892	19.762	18.792	19.905
19	29.355	30.862	30.857	31.482	30.649
20	6.181	0.631	0.771	0.980	3.169
21	31.736	27.538	22.098	20.509	25.857
22	31.833	27.764	22.741	21.052	26.194
23	34.470	32.255	32.393	31.838	32.755
24	27.746	25.320	21.586	20.650	23.996
25	30.216	30.142	26.237	24.589	27.904
26	20.123	18.924	13.763	15.361	17.237
27	31.402	26.795	24.208	21.814	26.296
28	29.973	27.894	24.003	25.343	26.902
29	29.966	27.525	24.618	25.643	27.015
30	29.860	32.634	30.199	29.803	30.646
31	15.447	9.754	13.325	11.967	12.792
32	32.234	25.686	24.249	18.890	25.708
33	19.313	18.175	16.090	11.954	16.622
34	31.060	26.630	23.550	21.756	25.990
35	30.071	27.136	24.316	23.089	26.292
36	29.439	30.119	26.938	23.384	27.597
37	28.470	23.456	20.084	14.413	22.203
38	10.893	4.367	3.239	8.895	7.539
39	34.381	31.994	32.021	31.557	32.507
40	22.689	23.255	25.439	27.057	24.672

Peptide 2					
**Ranking**	**Deviation (Å)**				**RMSD (Å)**
	P4	*P3*	*P2*	*P1*	
1	0.555	0.638	0.616	0.445	0.568
2	0.546	0.646	0.614	0.383	0.557
3	13.315	8.287	3.650	4.068	8.304
4	18.696	18.812	14.792	13.679	16.653
5	23.861	20.525	16.843	12.695	18.944
6	0.625	0.544	0.682	0.756	0.656
7	12.635	8.459	8.616	12.230	10.666
8	37.973	33.416	31.510	27.160	32.746
9	1.239	1.065	0.387	0.476	0.872
10	29.630	26.500	23.873	20.509	25.351
11	13.272	11.405	10.346	9.681	11.258
12	21.599	18.559	18.474	15.576	18.674
13	28.438	23.705	20.353	17.767	22.916
14	4.552	2.358	2.622	2.189	3.080
15	17.537	11.482	9.744	8.085	12.244
16	24.056	20.174	16.827	17.105	19.757
17	30.482	30.402	30.337	31.269	30.625
18	17.712	17.224	11.983	9.072	14.459
19	27.797	21.113	16.537	12.024	20.227
20	30.334	29.271	25.621	20.723	26.753
21	38.124	33.812	32.604	27.433	33.212
22	11.333	10.431	9.675	9.663	10.298
23	20.064	14.913	14.285	11.854	15.569
24	12.906	11.650	5.525	1.711	9.162
25	25.053	22.424	20.733	15.158	21.155
26	23.032	19.377	15.844	11.336	17.927
27	41.061	38.721	38.606	34.456	38.285
28	29.117	28.875	25.548	19.656	26.080
29	34.970	27.981	25.388	18.620	27.373
30	36.073	29.573	28.459	24.032	29.847
31	21.790	17.449	15.413	15.124	17.646
32	17.676	17.419	15.769	11.733	15.829
33	29.421	25.649	22.888	18.467	24.435
34	18.540	18.569	15.021	13.975	16.654
35	37.315	31.198	27.664	21.703	30.008
36	19.309	19.874	14.822	13.717	17.144
37	33.199	27.787	25.667	20.789	27.227
38	0.745	0.526	0.701	1.004	0.763
39	13.785	8.636	4.172	3.511	8.578
40	28.610	25.173	22.788	19.112	24.170

Peptide 3					
**Ranking**	**Deviation (Å)**				**RMSD (Å)**
	P4	*P3*	*P2*	*P1*	
1	11.898	7.809	1.303	6.506	7.851
2	23.181	16.380	10.563	4.065	15.279
3	21.375	17.295	14.571	11.297	16.552
4	20.953	15.725	19.831	18.326	18.811
5	29.626	27.569	22.286	20.224	25.216
6	16.900	17.037	16.321	17.637	16.980
7	15.259	8.595	3.408	4.180	9.162
8	34.033	32.487	30.714	26.474	31.056
9	27.685	26.231	23.336	22.612	25.052
10	40.175	35.169	31.132	25.347	33.402
11	27.886	26.856	27.162	26.899	27.204
12	15.469	9.031	3.766	3.816	9.349
13	17.990	16.932	13.953	12.753	15.554
14	20.601	20.131	19.251	20.858	20.219
15	23.907	17.900	18.867	20.651	20.459
16	15.972	10.648	4.278	2.913	9.941
17	18.113	18.376	15.694	13.355	16.510
18	28.422	23.714	23.635	21.246	24.393
19	24.405	21.608	18.356	15.256	20.200
20	15.195	8.625	3.605	3.970	9.138
21	29.275	27.440	22.585	20.852	25.273
22	27.867	27.591	23.014	22.175	25.294
23	23.167	19.542	17.246	15.462	19.073
24	22.629	18.852	17.926	13.329	18.483
25	44.617	41.349	39.960	36.192	40.642
26	44.700	41.208	39.550	35.504	40.376
27	22.662	16.161	10.385	4.213	15.003
28	23.907	18.682	15.821	13.244	18.346
29	22.391	15.797	13.637	11.300	16.314
30	32.843	30.974	32.785	32.223	32.215
31	22.878	21.267	20.096	21.467	21.450
32	24.833	22.145	19.383	14.554	20.583
33	23.664	23.184	27.114	28.236	25.641
34	22.422	22.828	23.759	26.057	23.808
35	24.498	25.178	27.726	29.359	26.762
36	22.776	16.403	10.496	3.984	15.115
37	13.372	10.599	8.571	8.791	10.511
38	15.813	14.702	13.782	14.830	14.799
39	35.034	35.680	34.739	32.458	34.499
40	35.552	33.192	32.097	29.156	32.580

41	21.999	20.491	16.194	14.350	18.520		41	21.853	16.738	17.953	14.406	17.941		41	17.951	18.080	16.320	13.311	16.527
42	29.654	29.093	24.277	23.912	26.865		42	24.896	20.004	16.804	11.509	18.939		42	29.979	25.653	23.150	20.742	25.115
43	29.046	25.650	22.535	22.643	25.110		43	35.004	31.941	30.647	27.939	31.486		43	15.998	9.970	4.367	2.993	9.790
44	17.333	12.783	7.424	1.329	11.410		44	23.816	18.964	13.084	7.623	17.001		44	19.676	17.881	15.871	14.505	17.096
45	29.782	30.492	27.242	24.646	28.135		45	11.978	8.283	8.695	12.517	10.540		45	33.535	34.767	33.957	30.967	33.337
46	2.977	2.419	3.897	4.738	3.618		46	34.266	27.318	24.928	18.255	26.810		46	18.557	19.095	17.362	16.749	17.965
47	23.776	17.510	14.650	7.907	16.949		47	28.640	26.898	28.080	27.713	27.840		47	20.068	18.848	15.834	14.661	17.490
48	18.357	12.796	6.210	1.131	11.625		48	9.581	9.353	8.949	8.396	9.081		48	9.365	3.661	9.214	15.616	10.367
49	28.459	25.941	22.612	22.700	25.047		49	19.449	18.647	13.456	9.572	15.801		49	18.545	17.061	14.079	12.946	15.818
50	28.902	27.319	27.084	26.634	27.498		50	22.239	18.108	16.285	15.849	18.295		50	28.853	30.215	29.093	26.412	28.677
51	16.950	12.619	7.377	1.610	11.220		51	9.066	9.094	10.232	15.039	11.133		51	16.569	9.669	10.577	9.529	11.945
52	18.827	18.476	15.425	15.600	17.155		52	20.329	17.853	15.926	13.272	17.042		52	15.162	8.471	3.408	4.337	9.111
53	22.520	20.156	14.798	11.219	17.736		53	24.660	19.423	20.589	17.433	20.695		53	14.336	10.410	5.476	2.397	9.349
54	17.635	12.795	7.484	2.020	11.563		54	26.931	23.806	22.163	19.554	23.268		54	22.998	19.905	15.490	10.235	17.817
55	6.025	1.049	1.360	0.606	3.147		55	28.746	28.796	25.555	20.933	26.205		55	19.235	19.814	16.979	12.397	17.353
56	34.977	34.766	32.347	32.463	33.661		56	18.878	17.061	16.788	12.578	16.489		56	11.885	7.879	1.406	6.353	7.837
57	23.732	23.631	23.334	23.154	23.464		57	21.119	18.738	18.268	18.582	19.210		57	20.482	15.989	11.219	7.434	14.631
58	19.883	18.503	15.127	15.560	17.383		58	27.911	32.009	27.883	25.605	28.446		58	10.578	4.250	9.663	13.476	10.062
59	20.828	19.422	15.715	11.400	17.234		59	29.455	26.473	23.649	20.074	25.153		59	24.914	22.227	19.383	15.904	20.877
60	24.589	23.006	24.288	24.204	24.029		60	22.431	21.058	22.253	21.483	21.813		60	22.795	15.683	9.992	3.958	14.841
61	21.450	18.219	16.360	13.440	17.609		61	17.976	15.879	12.612	9.718	14.394		61	22.172	22.355	24.973	26.153	23.974
62	19.482	21.221	15.798	14.975	18.053		62	0.762	0.717	0.910	0.753	0.789		62	24.748	21.858	22.862	20.350	22.511
63	28.894	28.875	27.866	26.015	27.937		63	36.155	30.113	28.964	25.422	30.410		63	12.526	7.426	8.893	10.875	10.117
64	22.148	16.510	14.849	15.612	17.517		64	26.073	22.619	23.296	23.210	23.837		64	14.472	11.588	9.870	8.401	11.310
65	30.362	33.225	30.485	27.293	30.414		65	29.145	25.869	23.702	19.528	24.808		65	18.018	17.833	12.184	10.912	15.085
66	30.776	30.913	26.655	24.429	28.329		66	0.673	0.699	0.662	0.965	0.760		66	18.800	18.891	15.754	12.679	16.728
67	10.358	10.700	16.707	21.025	15.354		67	24.837	18.770	18.748	16.523	19.960		67	25.175	20.676	17.202	13.105	19.551
68	22.208	21.067	18.147	15.014	19.312		68	28.037	22.982	24.016	19.861	23.903		68	20.348	15.872	16.241	13.253	16.624
69	29.541	25.938	22.445	23.849	25.583		69	0.954	0.818	0.796	0.585	0.800		69	16.008	11.465	11.262	13.937	13.311
70	28.591	24.011	21.080	15.032	22.717		70	29.292	27.457	25.633	24.716	26.832		70	23.274	19.565	17.454	15.895	19.247
71	22.100	20.028	19.769	19.011	20.260		71	16.651	13.998	13.291	12.763	14.255		71	21.379	18.088	16.459	13.842	17.655
72	18.897	14.300	9.538	10.896	13.886		72	23.389	17.825	15.693	12.069	17.725		72	13.018	7.663	6.783	9.846	9.633
73	30.024	27.437	23.294	22.186	25.928		73	23.414	20.060	16.998	15.048	19.144		73	22.374	17.668	12.173	13.009	16.809
74	16.973	13.134	7.715	1.772	11.437		74	35.819	30.127	29.447	25.548	30.457		74	22.923	19.600	15.574	10.362	17.745
75	21.811	17.673	14.451	12.799	17.035		75	0.616	0.929	1.324	1.358	1.100		75	15.969	10.744	4.330	2.964	9.974
76	22.697	16.972	16.652	17.556	18.633		76	36.136	37.749	34.738	32.044	35.229		76	22.043	20.931	17.130	13.422	18.692
77	14.722	11.995	11.241	13.165	12.848		77	35.228	31.906	31.795	28.447	31.934		77	29.216	25.742	23.515	18.497	24.552
78	20.211	19.284	16.563	15.319	17.954		78	33.054	34.975	33.758	31.149	33.263		78	11.896	8.710	13.649	15.934	12.821
79	7.662	8.211	13.077	18.129	12.508		79	19.717	16.627	16.026	16.071	17.178		79	21.040	17.156	14.913	15.772	17.379
80	23.075	23.584	26.542	27.221	25.170		80	28.899	29.363	26.879	25.853	27.786		80	12.987	9.846	7.451	8.250	9.864
81	2.517	2.559	3.597	4.657	3.447		81	30.093	29.367	24.445	21.578	26.604		81	24.373	21.063	19.039	19.258	21.042
82	23.282	23.631	22.898	25.139	23.753		82	29.200	28.013	23.478	21.799	25.806		82	21.434	14.656	16.151	15.620	17.169
83	22.426	23.086	25.967	26.888	24.664		83	13.186	11.383	10.268	9.852	11.247		83	14.957	8.277	3.525	4.132	8.969
84	0.869	1.315	1.760	2.971	1.898		84	38.157	35.915	31.511	25.938	33.210		84	40.267	35.113	31.168	25.455	33.443
85	33.064	32.368	27.822	25.192	29.789		85	20.008	20.531	15.623	14.318	17.825		85	21.326	16.886	15.093	15.389	17.353
86	15.788	10.397	13.368	12.356	13.122		86	22.414	21.413	20.852	20.397	21.282		86	31.943	30.820	28.342	24.297	28.999
87	18.188	12.302	5.782	0.741	11.359		87	22.991	16.185	13.104	6.818	15.881		87	12.227	8.545	10.873	10.704	10.669
88	26.414	25.839	21.916	20.254	23.748		88	23.757	19.784	15.347	10.038	17.973		88	12.386	7.134	8.384	13.237	10.605
89	22.957	18.111	14.596	12.203	17.443		89	35.981	29.233	25.258	18.578	27.984		89	34.346	35.043	33.712	32.345	33.876
90	29.125	26.231	23.740	21.325	25.272		90	26.829	23.477	24.243	23.460	24.541		90	12.972	10.037	10.174	9.871	10.839
91	18.260	12.371	5.664	0.985	11.397		91	14.824	13.455	17.307	18.009	16.005		91	24.566	21.802	18.858	15.277	20.419
92	18.579	17.262	14.294	13.134	15.969		92	29.372	26.222	23.826	20.238	25.137		92	21.103	17.107	15.082	13.656	16.970
93	10.793	4.126	3.746	9.602	7.742		93	22.013	22.121	22.860	25.346	23.124		93	27.402	24.602	21.427	16.428	22.831
94	19.196	17.789	15.619	11.840	16.348		94	34.660	28.950	28.664	23.895	29.292		94	13.994	14.006	13.201	14.261	13.871
95	29.126	30.097	26.909	23.291	27.481		95	13.801	8.487	11.597	11.875	11.597		95	23.152	17.972	16.728	12.874	18.059
96	22.880	17.966	12.117	12.871	17.020		96	22.596	19.734	16.859	13.379	18.461		96	13.646	10.899	10.554	8.872	11.125
97	39.615	36.685	32.698	28.006	34.528		97	0.880	1.168	0.999	0.996	1.016		97	16.995	17.135	16.648	17.737	17.133
98	35.822	36.519	34.517	32.731	34.927		98	25.711	22.574	23.292	22.684	23.600		98	21.506	16.417	18.341	14.722	17.924
99	27.975	26.127	24.801	22.370	25.400		99	26.936	24.052	25.067	23.187	24.850		99	23.718	21.634	22.663	21.199	22.325
100	18.301	12.586	6.158	1.055	11.537		100	17.729	13.154	8.905	4.514	12.114		100	18.771	17.789	15.972	14.502	16.839

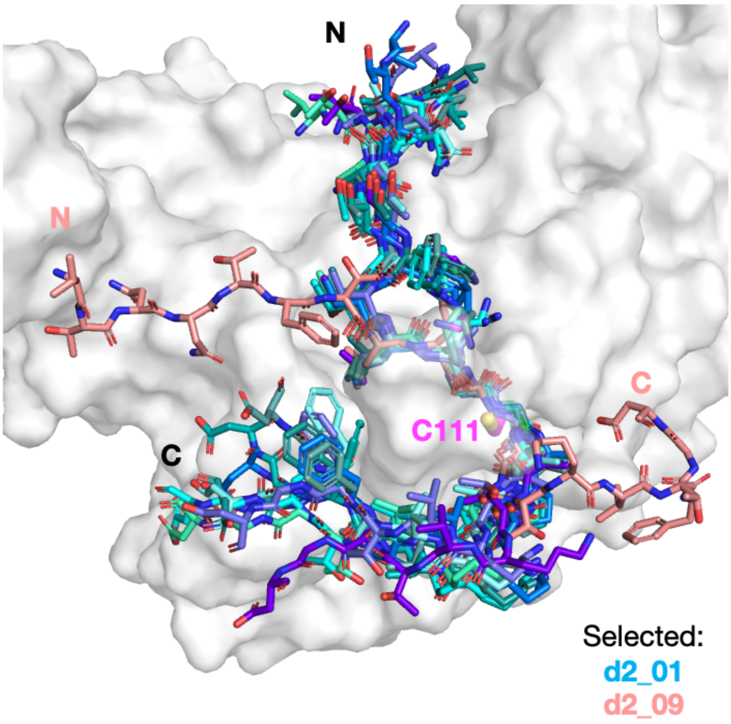

Figure S2: Docked conformations of peptide **2** that passed the applied filter, *i.e.*, the four Cα atoms of P4-P1 have to be within 2 Å of the corresponding Cα atoms of VIR251.[3] The poses include ranked solutions 01 (cyan), 02 (green-cyan), 06 (aquamarine), 09 (salmon), 38 (teal), 62 (deep teal), 66 (light teal), 69 (marine), 75 (slate), and 97 (purple blue). Except for d2_09, which adopts a unique extended conformation, d2_01 is representative of all the remaining poses.

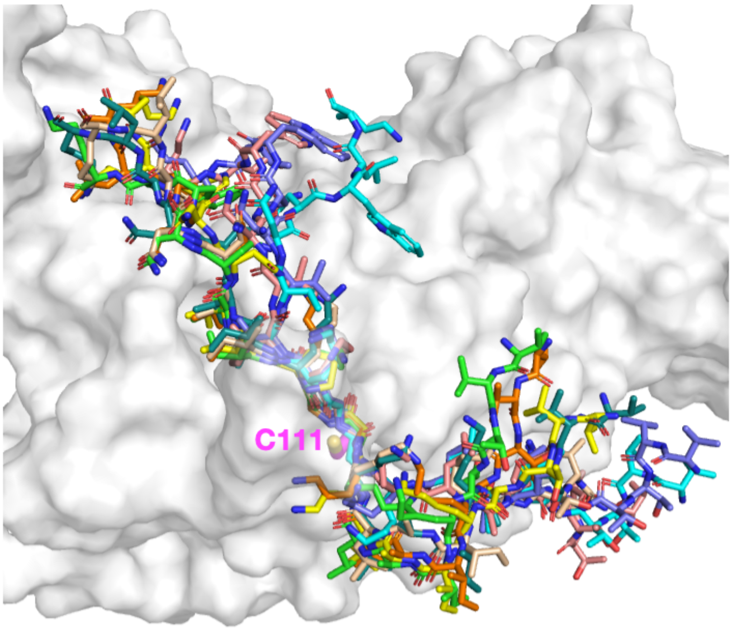

Figure S3: Docked reverse-bound poses of peptide **3**, which position the original P2-Gly residue in the S1 subsite, P1-Gly in the S2 subsite, P1′-Lys in the S3 subsite, and P2′-Ile in the S4 subsite. The poses include ranked solutions 07 (orange), 12 (green), 16 (cyan), 20 (yellow), 43 (salmon), 52 (teal), 75 (slate), and 83 (wheat). The highest ranked solution among them, d3_07, was selected as being representative and was used for subsequent modeling.

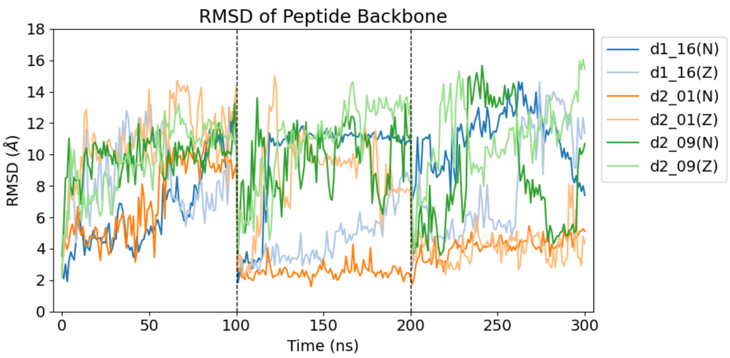

Figure S4: RMSD of peptide backbone atoms (N, Cα, C) in the combined 3 × 100 ns molecular dynamics (MD) trajectories (fitted based on PL^{pro} backbone) initiated from the d1_16, d2_01, and d2_09 poses in either the neutral (N) or zwitterionic (Z) state of Cys111-His272, relative to their corresponding starting peptide poses.

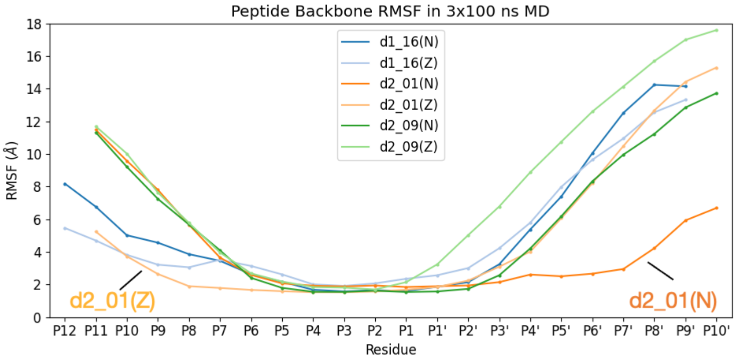

Figure S5: Per-residue root mean square fluctuation (RMSF) of peptide backbone atoms in the combined 3 × 100 ns MD (fitted based on PL^{pro} backbone) initiated from the d1_16, d2_01, and d2_09 poses in either the N or Z state of Cys111-His272. Note that the C-terminal NH_2 group is treated as a separate residue.

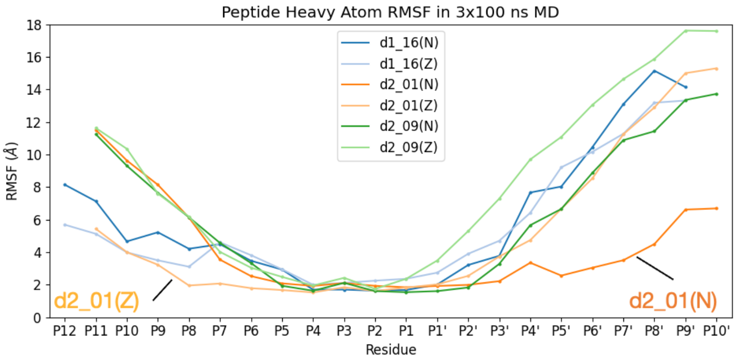

Figure S6: Per-residue RMSF of peptide non-hydrogen atoms in the combined 3 × 100 ns MD (fitted based on PL^{pro} backbone) initiated from the d1_16, d2_01, and d2_09 poses in either the N or Z state of Cys111-His272.

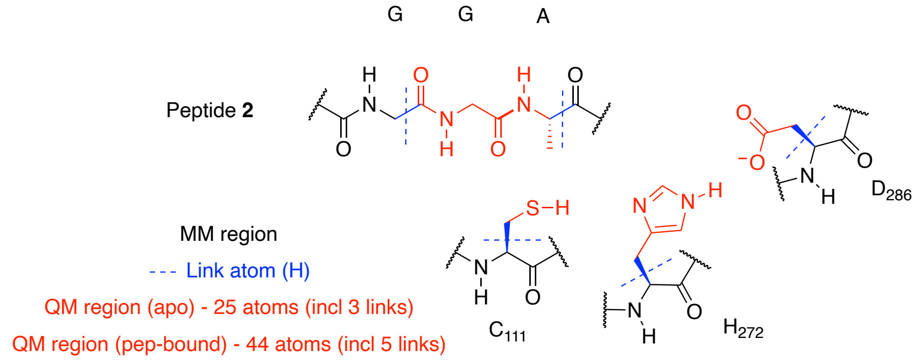

Figure S7: Definition of the quantum mechanical (QM) region in the quantum mechanics/molecular mechanics-umbrella sampling (QM/MM-US) calculations for proton transfer processes in the Cys111-His272-Asp286 catalytic triad.

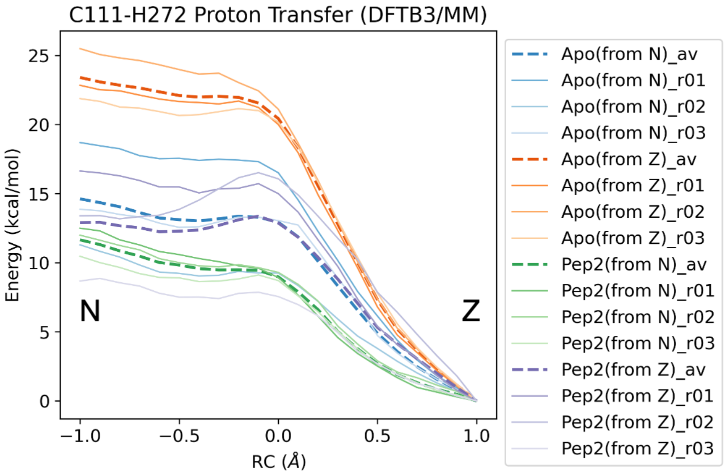

Figure S8: DFTB3/MM-US free energy profiles from the weighted histogram analysis method (WHAM)[4] for the proton transfer between Cys111 and His272, with reaction coordinate (RC) = -1 being the N state and +1 being the Z state. The energy profiles averaged from three replicas are shown as dotted lines.

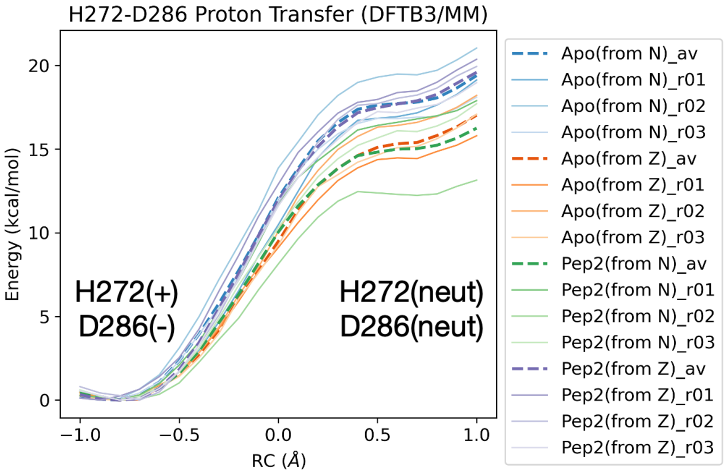

Figure S9: WHAM[4]-derived DFTB3/MM-US free energy profiles for the proton transfer between His272 and Asp286, between RC = -1 (doubly protonated His272 and deprotonated Asp286) and +1 (neutral His272 and neutral Asp286). The energy profiles averaged from three replicas are shown as dotted lines.

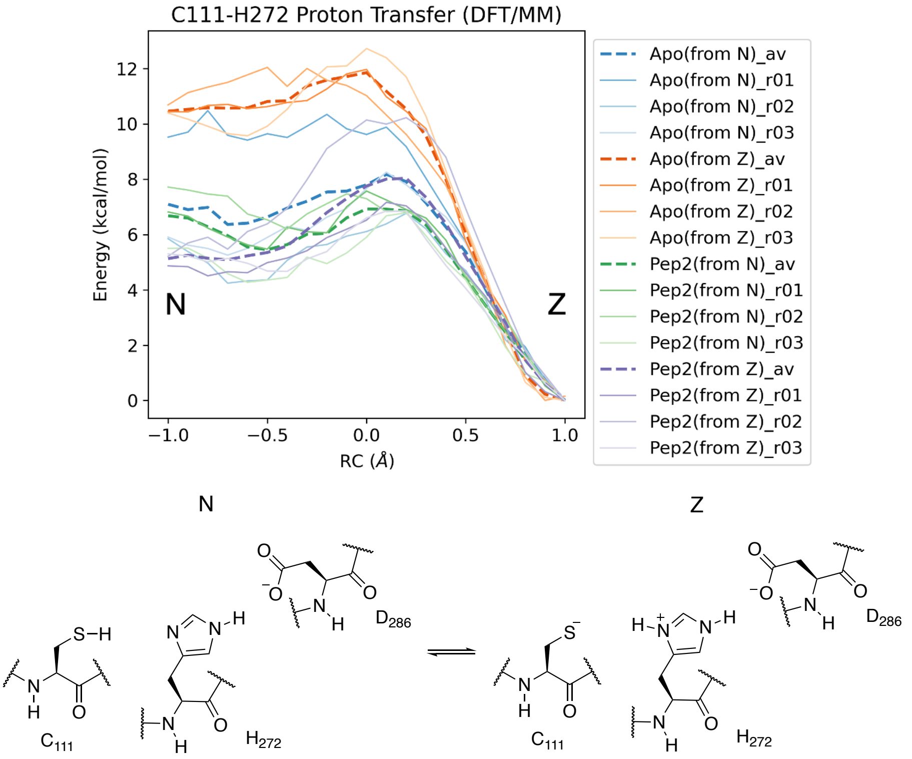

Figure S10: WHAM[4]-derived PBE0-D3BJ/6-31G(d)/MM-US free energy profiles for the proton transfer between Cys111 and His272, with RC = -1 being the N state and +1 being the Z state. The energy profiles averaged from three replicas are shown as dotted lines.

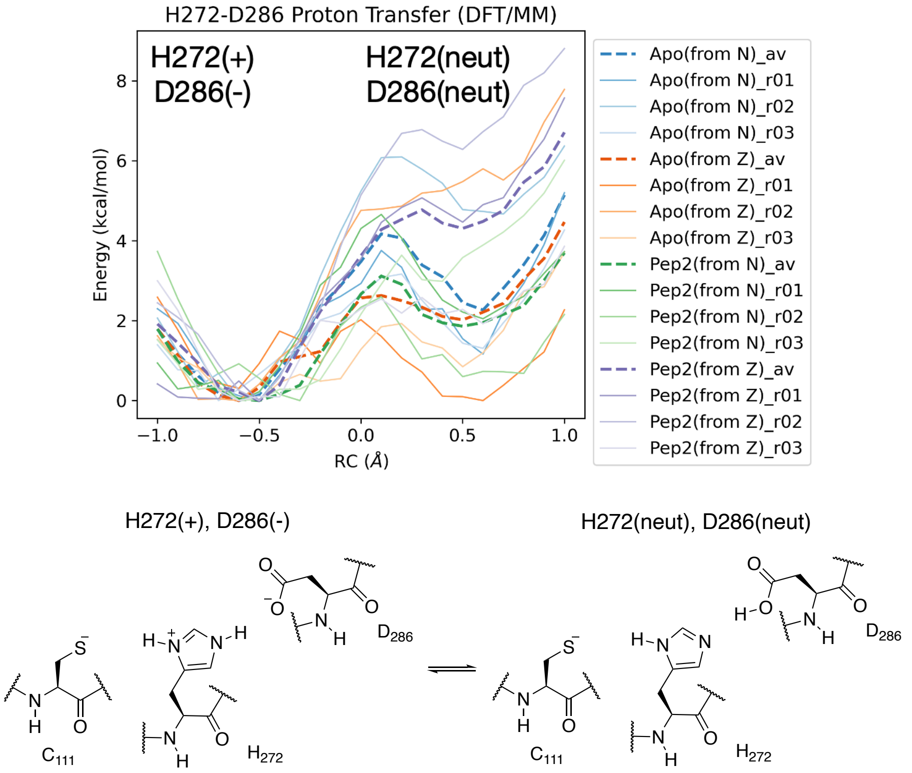

Figure S11: WHAM[4]-derived PBE0-D3BJ/6-31G(d)/MM-US free energy profiles for the proton transfer between His272 and Asp286, between RC = -1 (doubly protonated His272 and deprotonated Asp286) and +1 (neutral His272 and neutral Asp286). The energy profiles averaged from three replicas are shown as dotted lines.

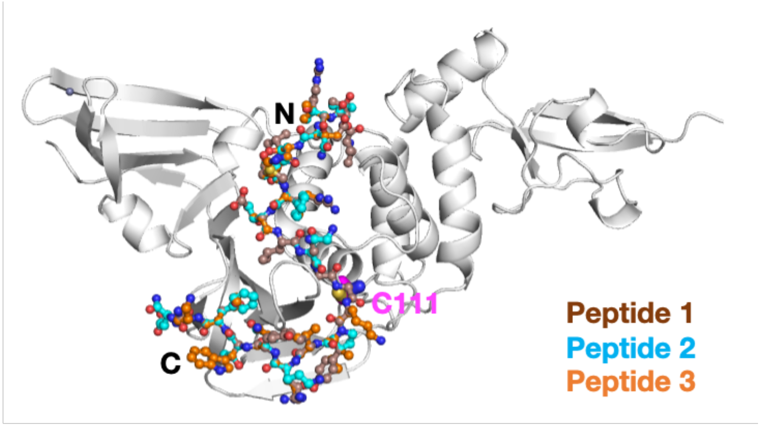

Figure S12: Starting conformations of PL^{pro} complexed with each of the three nsp oligopeptides **1**, **2**, and **3**, based on the d2_01 conformation.

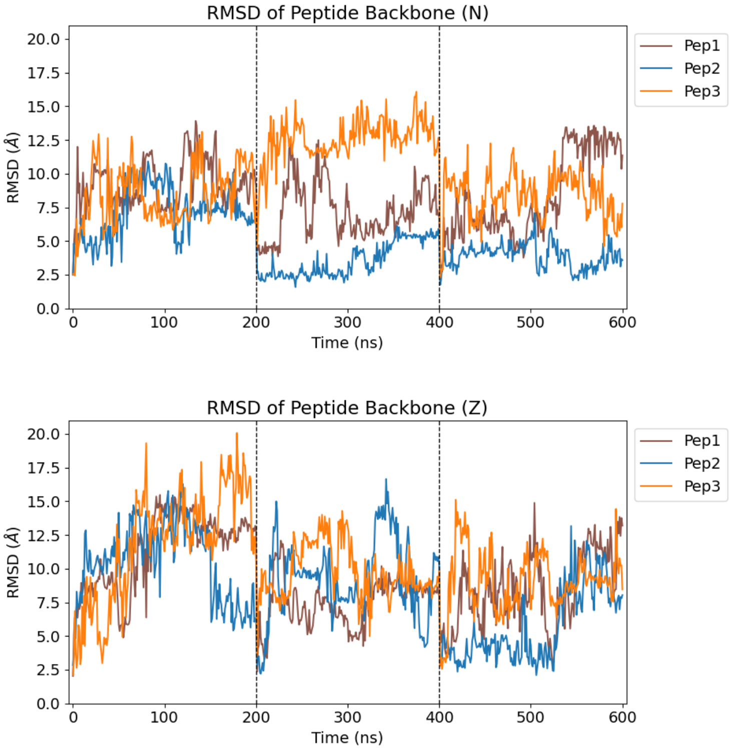

Figure S13: RMSD of peptide backbone atoms in the combined 3 × 200 ns MD trajectories (fitted based on PL^{pro} backbone) for the three nsp peptides initiated from the d2_01 conformation, in the (top) N and (bottom) Z states of Cys111-His272, relative to their corresponding starting conformations.

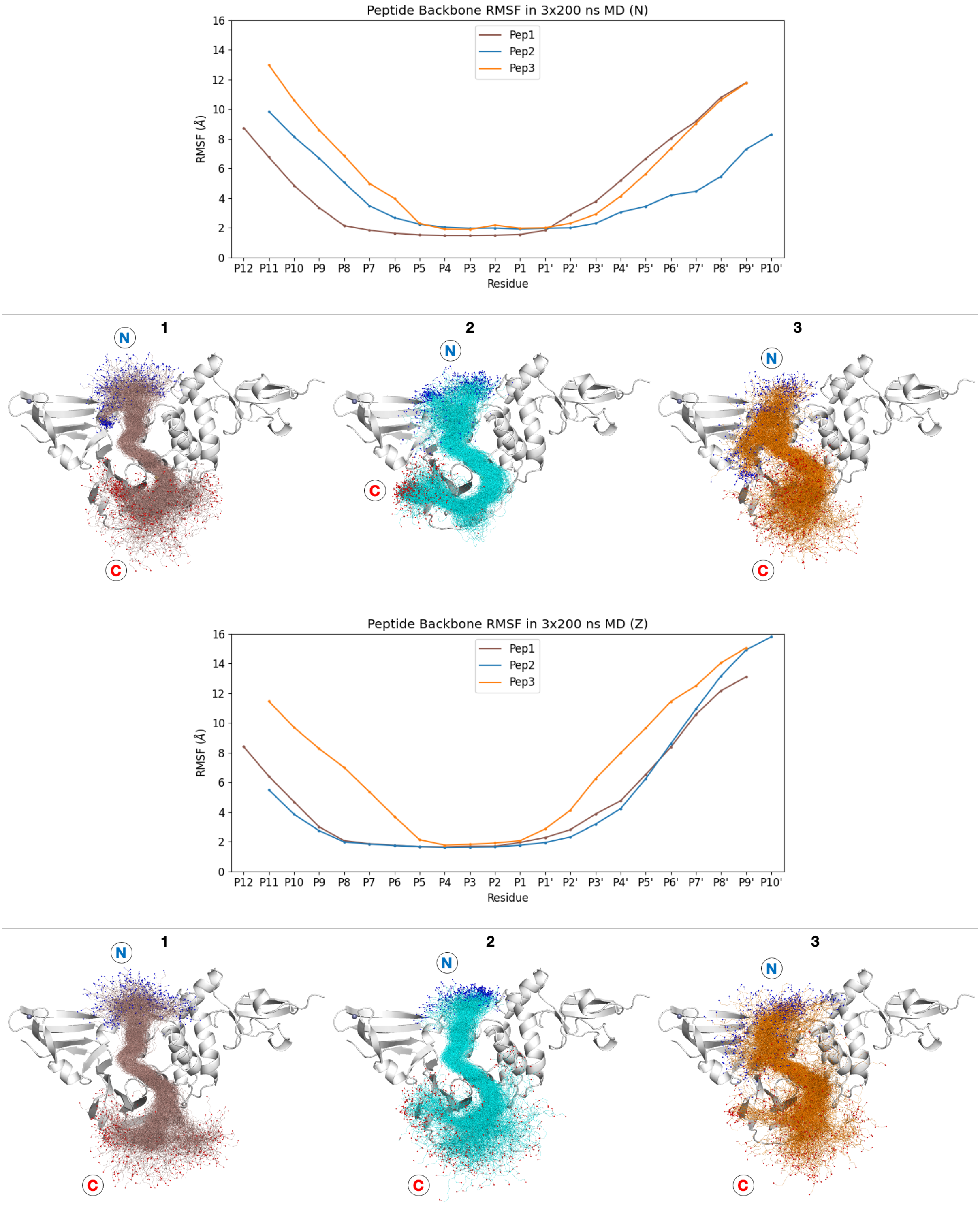

Figure S14: Per-residue RMSF of peptide backbone atoms in the combined 3 × 200 ns MD (fitted based on PL^{pro} backbone) for the three nsp peptides initiated from the d2_01 conformation, and views of the peptide backbone configurations sampled over the course of MD simulations (frames extracted every ns), in the (top) N and (bottom) Z states of Cys111-His272. Note that the C-terminal NH_2 group is treated as a separate residue in the RMSF plots. The N- and C-terminals are shown as blue and red spheres, respectively.

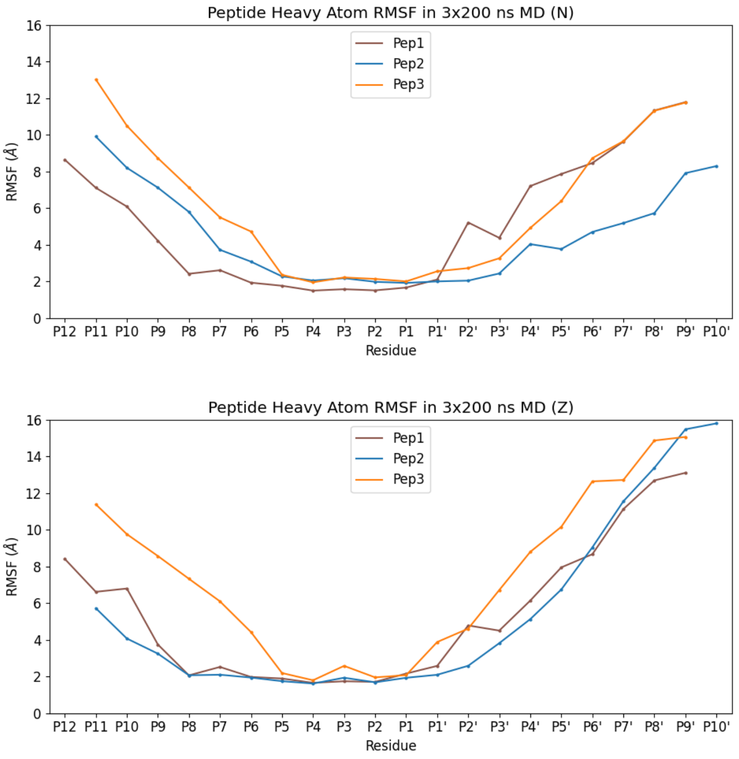

Figure S15: Per-residue RMSF of peptide non-hydrogen atoms in the combined 3 × 200 ns MD (fitted based on PL^{pro} backbone) for the three nsp peptides initiated from the d2_01 conformation, in the (top) N and (bottom) Z states of Cys111-His272.

Table S2: PL^{pro}-peptide hydrogen bonds (HBs) of significance (occurrence ≥ 25% out of 600 frames analyzed every ns) observed over the combined 3 × 200 ns MD for each of the three nsp peptides **1**, **2**, and **3**, all initiated from the d2_01 conformation, in the (left) N and (right) Z states of Cys111-His272. HB occurrences involving equivalent carboxylate oxygens (in Asp and Glu sidechains) are combined, while salt bridges involving carboxylate oxygens and Arg sidechain guanidino group are combined and halved to avoid double counting. Peptide residues are italicized, while PL^{pro} residues are not. Outside the core P5-P1′ region (see **Figure 3a**), N-terminal HBs are in blue and C-terminal HBs are in red.

Donor	Acceptor	%		Donor	Acceptor	%
Pep 1 (N)	P5-P1′=8-13			Pep 1 (Z)		
GLY12N	GLY271O	96		*GLU8N*	ASP164OD	97
GLY11N	GLY163O	95		*ASN10ND2*	TYR268O	96
GLU8N	ASP164OD	94		*GLY11N*	GLY163O	95
GLY163N	*GLY11O*	93		*LEU9N*	ASP164OD	83
ASN10ND2	TYR268O	91		GLY271N	*ASN10O*	80
LEU9N	ASP164OD	80		GLY163N	*GLY11O*	80
ARG166NH	*GLU8OE*	80		ARG166NH	*GLU8OE*	80
ASN10N	TYR264OH	74		*ASN10N*	TYR264OH	79
GLY271N	*ASN10O*	74		*GLY12N*	GLY271O	76
TRP106NE1	*GLY12O*	59		*ALA13N*	GLY271O	48
ALA13N	GLY271O	36		*THR15OG1*	ASP286OD	35
ARG3NH	GLU203OE	32		*LEU5N*	TYR171OH	28
ARG7NH	GLU161OE	25		*ARG3NH*	GLU203OE	25
Pep 2 (N)	P5-P1′=7-12			Pep 2 (Z)		
GLY11N	GLY271O	95		*THR7N*	ASP164OD	100
TRP106NE1	*GLY11O*	95		*GLY10N*	GLY163O	94
GLY163N	*GLY10O*	92		*LEU8N*	ASP164OD	86
GLY10N	GLY163O	92		GLY271N	*LYS9O*	80
THR7N	ASP164OD	70		GLY163N	*GLY10O*	74
GLY266N	*VAL16O*	69		*LYS9N*	TYR264OH	68
THR265OG1	*THR14O*	68		*GLY11N*	GLY271O	66
LEU8N	ASP164OD	64		*ASN4ND2*	GLN174OE1	58
GLY271N	*LYS9O*	56		*ASN4N*	TYR171OH	45
LYS9N	TYR264OH	44		HIP272ND1	*GLY11O*	42
GLN250N	*GLY19O*	41		*ALA12N*	GLY271O	39
ALA12N	GLY271O	36		ASN109ND2	*GLY11O*	35
ARG166NH	*THR7OG1*	33		GLN174NE2	*ASN4OD1*	33
GLN250NE2	*PHE18O*	28		ARG166NH	*THR7OG1*	29
ASN4ND2	GLN174OE1	26		GLN174NE2	*THR2O*	27
Pep 3 (N)	P5-P1′=7-12			Pep 3 (Z)		
TRP106NE1	*GLY11O*	96		*GLY10N*	GLY163O	88
GLY163N	*GLY10O*	94		*GLY11N*	GLY271O	72
GLY11N	GLY271O	94		GLY163N	*GLY10O*	58
GLY10N	GLY163O	92		GLY271N	*LYS9O*	38
GLY271N	*LYS9O*	60		*LYS12N*	GLY271O	34
LYS9N	TYR268O	35		*LYS9N*	TYR264OH	32
LEU8N	ASP164OD	28		*LYS9N*	TYR268O	28
ALA7N	ASP164OD	28				

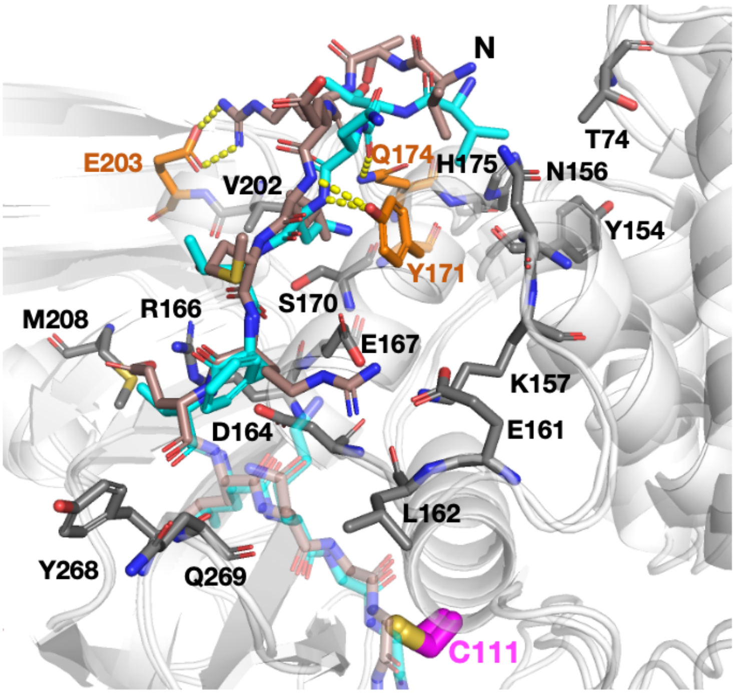

Figure S16: View of clustering-derived representative structures of Z-state PL^{pro} (white cartoon) complexed with peptides **1** (brown) or **2** (cyan), with a focus on the interactions in the N-terminal region outside the consensus P4-P1 sequence. PL^{pro} residues involved in HBs (yellow dotted lines; see **Table S2**; not all significant HBs are present in these snapshots) are shown as orange sticks. Other residues in close contact (within 4 Å) with the peptide residues N-terminal of P4 are in grey. The active site Cys111 is shown as magenta sticks. Clustering was performed using a 3 Å RMSD cut-off on the peptide non-hydrogen atoms using gmx cluster (gromos algorithm).[5,6] Out of 601 frames extracted every ns from the combined 3 × 200 ns MD (including $t = 0$), the number of (multimembered) clusters obtained for peptides **1**, **2**, and **3** are respectively 291 (81), 233 (76), and 361 (77).

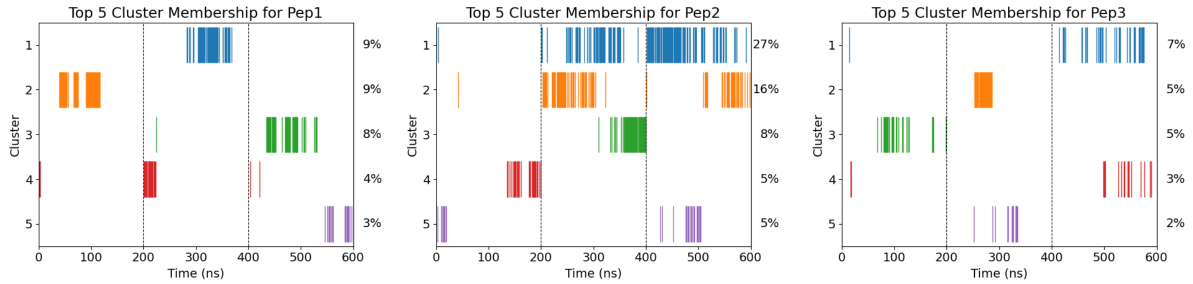

Figure S17: Cluster membership of frames extracted every ns from the combined 3 × 200 ns MD (fitted using PL^{pro} backbone) of each of the three PL^{pro}-peptide (N state) complexes, with clustering performed using a 3 Å RMSD cut-off on the peptide backbone atoms (N, Cα, C) using gmx cluster (gromos algorithm).[5,6] Only the 5 clusters with the highest occupancy percentages are shown. Out of 601 frames, the number of (multimembered) clusters obtained for peptides **1**, **2**, and **3** are respectively 157 (73), 66 (41), and 201 (77).

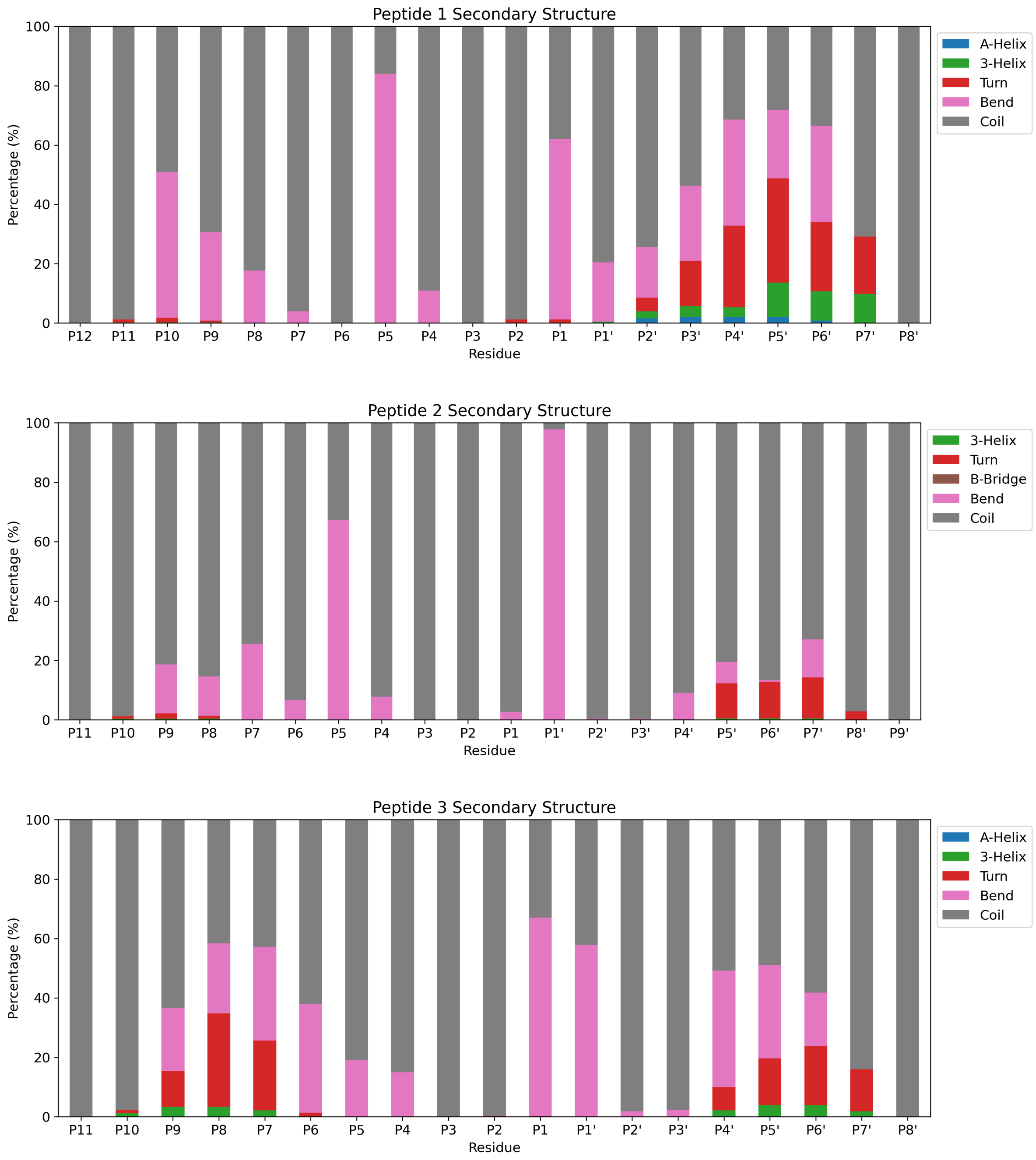

Figure S18: Proportion of secondary structure adopted by every peptide residue over the combined 3 × 200 ns MD of PL^{pro}-peptide (N state) complexes, assigned by DSSP (version 2.0.4).[7,8] Frames were analyzed every ns.

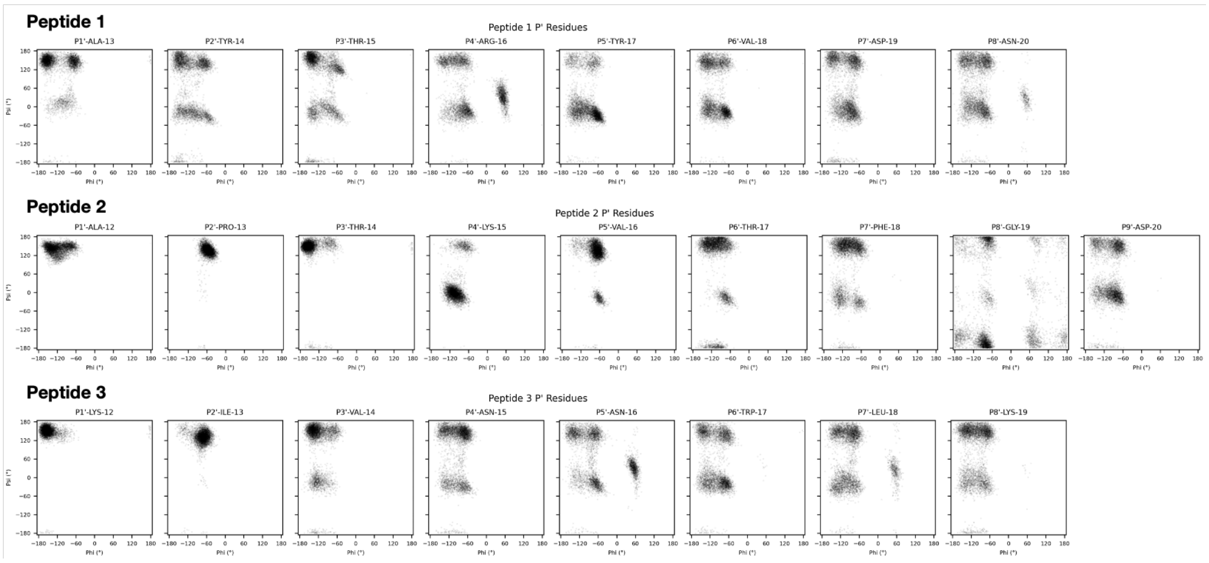

Figure S19: Ramachandran plots of every peptide P′ residue over the combined 3 × 200 ns MD of PL^{pro}-peptide (N state) complexes. Frames were analyzed every 0.1 ns.

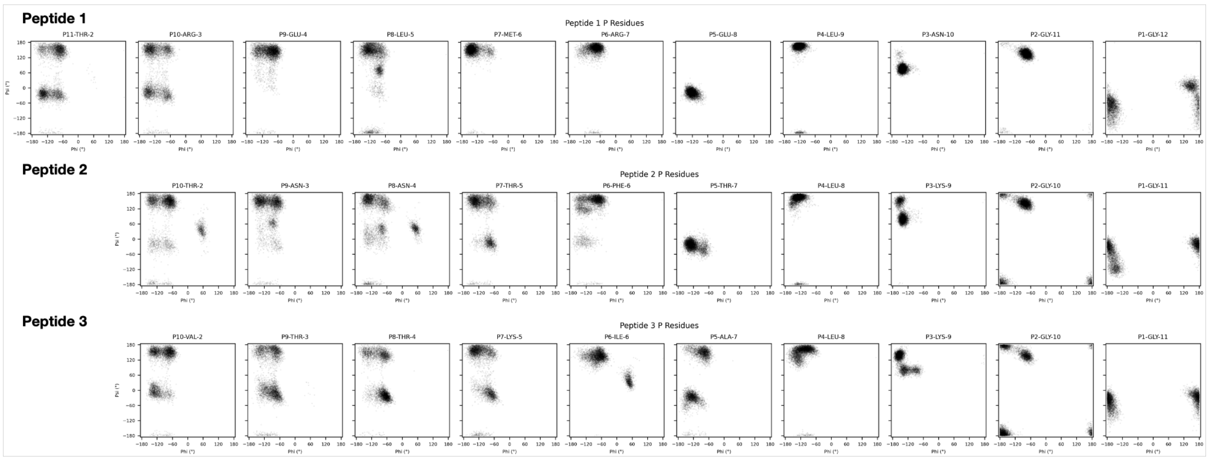

Figure S20: Ramachandran plots of every peptide P residue over the combined 3 × 200 ns MD of PL^{pro}-peptide (N state) complexes. Frames were analyzed every 0.1 ns.

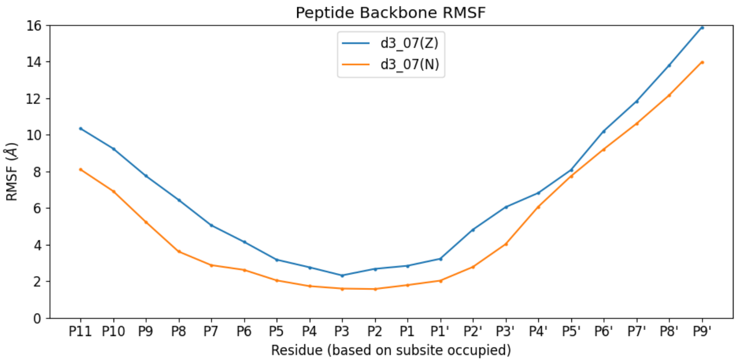

Figure S21: Per-residue RMSF of peptide backbone atoms in the combined 3 × 200 ns MD (fitted based on PL^{pro} backbone) for peptide **3** initiated from the d3_07 conformation, in the N (orange) and Z (blue) states of Cys111-His272. Note that the C-terminal NH_2 cap is treated as a separate residue (P11).

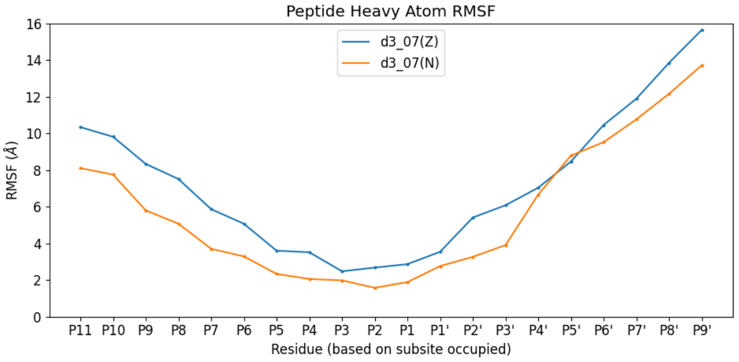

Figure S22: Per-residue RMSF of peptide non-hydrogen atoms in the combined 3 × 200 ns MD (fitted based on PL^{pro} backbone) for peptide **3** initiated from the d3_07 conformation, in the N (orange) and Z (blue) states of Cys111-His272.

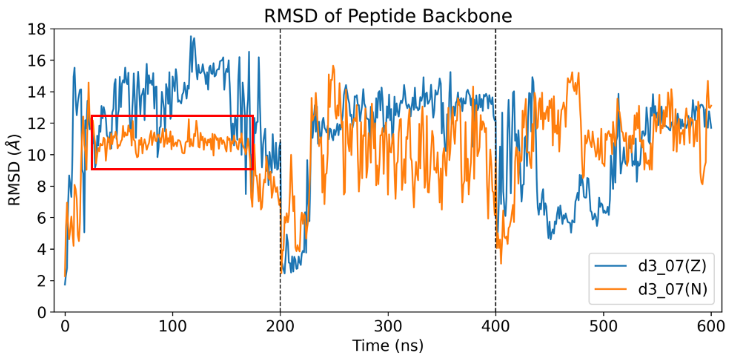

Figure S23: RMSD of peptide backbone atoms in the combined 3 × 200 ns MD trajectories (fitted based on PL^{pro} backbone) for peptide **3** initiated from the d3_07 conformation, in the N (orange) and Z (blue) states of Cys111-His272, relative to the starting conformation. The period of stable binding in the N state is indicated by a red box.

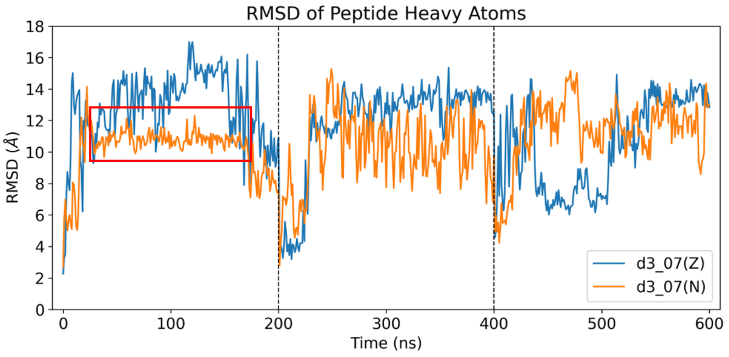

Figure S24: RMSD of peptide non-hydrogen atoms in the combined 3 × 200 ns MD trajectories (fitted based on PL^{pro} backbone) for peptide **3** initiated from the d3_07 conformation, in the N (orange) and Z (blue) states of Cys111-His272, relative to the starting conformation. The period of stable binding in the N state is indicated by a red box.

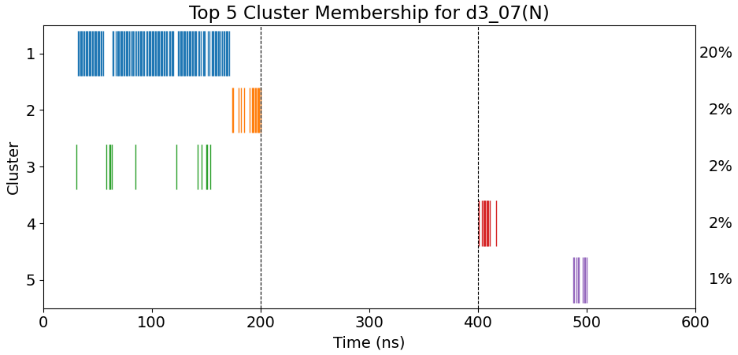

Figure S25: Cluster membership of frames extracted every ns from the combined 3 × 200 ns MD (fitted using PL^{pro} backbone) of PL^{pro} complexed with d3_07 (N state), with clustering performed using a 3 Å RMSD cut-off on the peptide non-hydrogen atoms using gmx cluster (gromos algorithm).[5,6] Only the 5 clusters with the highest occupancy percentages are shown. Cluster 1, which has the highest population, corresponds to the period of stable binding shown in **Figures S23-S24**. The complex structure at t = 159 ns is determined to be the representative middle structure for cluster 1, as defined by the smallest average RMSD to all other structures in the same cluster. Out of 601 frames, the number of (multimembered) clusters obtained is 344 (63).

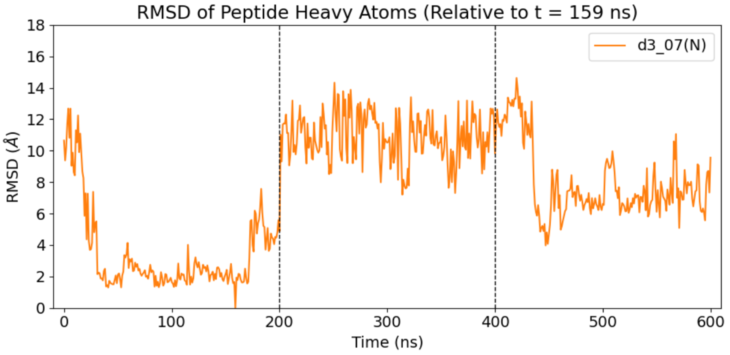

Figure S26: RMSD of peptide non-hydrogen atoms in the combined 3 × 200 ns MD trajectories (fitted based on PL^{pro} backbone) for peptide **3** initiated from the d3_07 conformation in the N state, relative to the t = 159 ns frame which is representative of cluster 1 (see **Figure S25**).

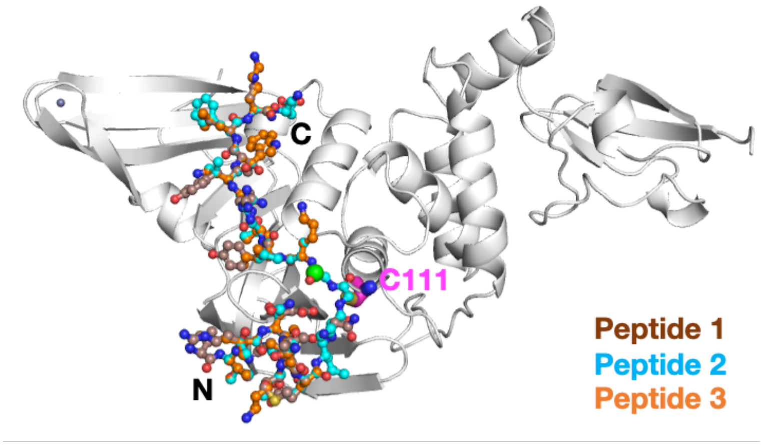

Figure S27: Starting conformations of PL^{pro} complexed with the three nsp oligopeptides **1**, **2**, and **3** in the reverse active site binding mode.

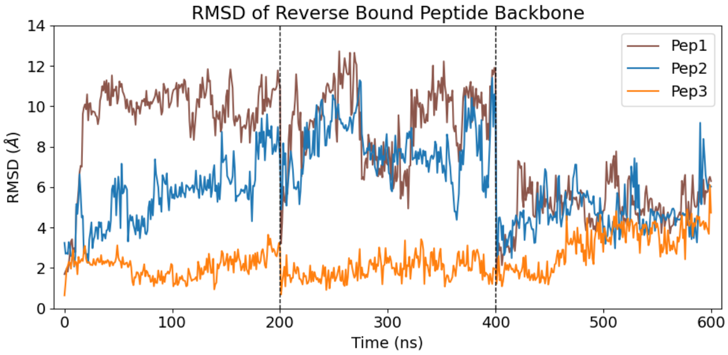

Figure S28: RMSD of peptide backbone atoms in the combined 3 × 200 ns MD trajectories (fitted based on PL^{pro} backbone) for the three reverse-bound nsp peptides, in the Z state of Cys111-His272, relative to their corresponding starting conformations.

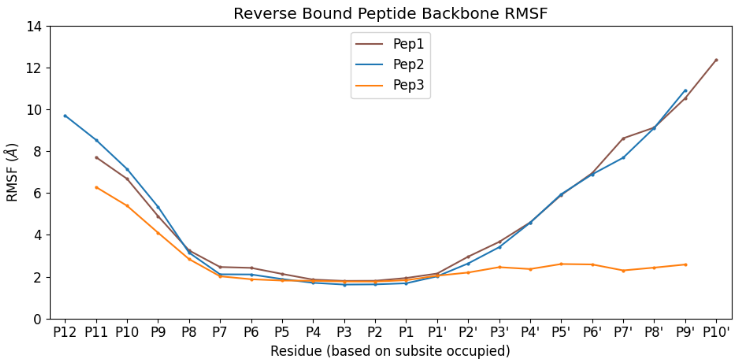

Figure S29: Per-residue RMSF of peptide backbone atoms in the combined 3 × 200 ns MD (fitted based on PL^{pro} backbone) for the three reverse-bound nsp peptides, in the Z state of Cys111-His272. Note that the C-terminal NH_2 cap is treated as a separate residue (P12 for peptide **2**; P11 for peptides **1** and **3**).

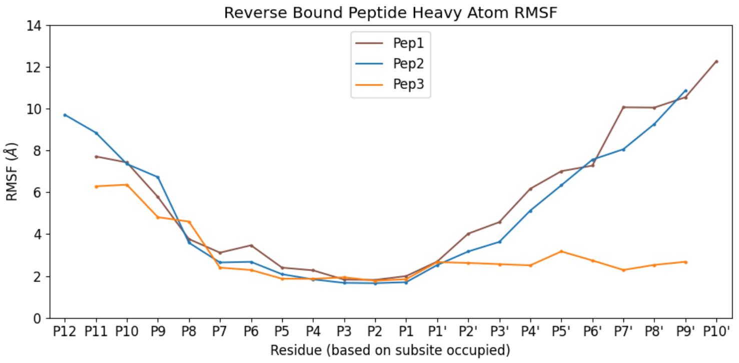

Figure S30: Per-residue RMSF of peptide non-hydrogen atoms in the combined 3 × 200 ns MD (fitted based on PL^{pro} backbone) for the three reverse-bound nsp peptides, in the Z state of Cys111-His272.

Table S3: PL^{pro}-peptide HBs of significance (occurrence ≥ 25% out of 600 frames analyzed every ns) observed over the combined 3 × 200 ns MD for each of the three reverse-bound nsp peptides, in the Z state of Cys111-His272. HB occurrences involving equivalent carboxylate oxygens (in Asp and Glu sidechains) are combined, while salt bridges involving carboxylate oxygens and Arg sidechain guanidino group are combined and halved to avoid double counting. Peptide residues are italicized, while PL^{pro} residues are not. HBs in the S and S′ subsites that are outside the S4-S1 subsites are in blue and red respectively.

Donor	Acceptor	%		Donor	Acceptor	%		Donor	Acceptor	%
Pep 1 (Z)	Res 10-13 in S1-S4			Pep 2 (Z)	Res 10-13 in S1-S4			Pep 3 (Z)	Res 10-13 in S1-S4	
ALA12N	GLY163O	94		*ALA12N*	GLY163O	85		ASN267ND2	*VAL1O*	95
GLY271N	*GLY11O*	87		ARG166NH	*LYS15O*	69		ASN267ND2	*THR3OG1*	94
TYR112N	*GLY10O*	58		*THR14OG1*	ASP164OD	68		*LYS12N*	GLY163O	93
GLY11N	GLY271O	52		TYR112N	*GLY10O*	51		GLY271N	*GLY11O*	91
ARG6NH	ASP286OD	42		*LYS15NZ*	GLU167OE	51		*THR3OG1*	GLN269OE1	81
TYR13N	GLN269O	36		*GLY10N*	GLY271O	35		*ILE13N*	GLN269O	71
ARG15NH	GLU167OE	35		ARG166NE	*THR14OG1*	34		TYR112N	*GLY10O*	66
THR14OG1	ASP164OD	32		TYR273OH	*ALA12O*	33		TYR273OH	*LYS12O*	48
TYR273OH	*ALA12O*	32		GLY271N	*GLY11O*	33		ARG166NH	*ASN15O*	46
HIP272ND1	*ASN9O*	30		*GLY11N*	GLY271O	33		*LYS12NZ*	ASP164OD	44
ARG166NE	*THR14OG1*	26		ARG166NH	*ASP20OD*	30		HIP272ND1	*LYS9O*	34
								ARG166NE	*ASN15O*	33
								GLY11N	GLY271O	30
								LYS19NZ	GLU203OE	30
								LYS12NZ	GLU167OE	29
								ASN109ND2	*LYS9O*	26

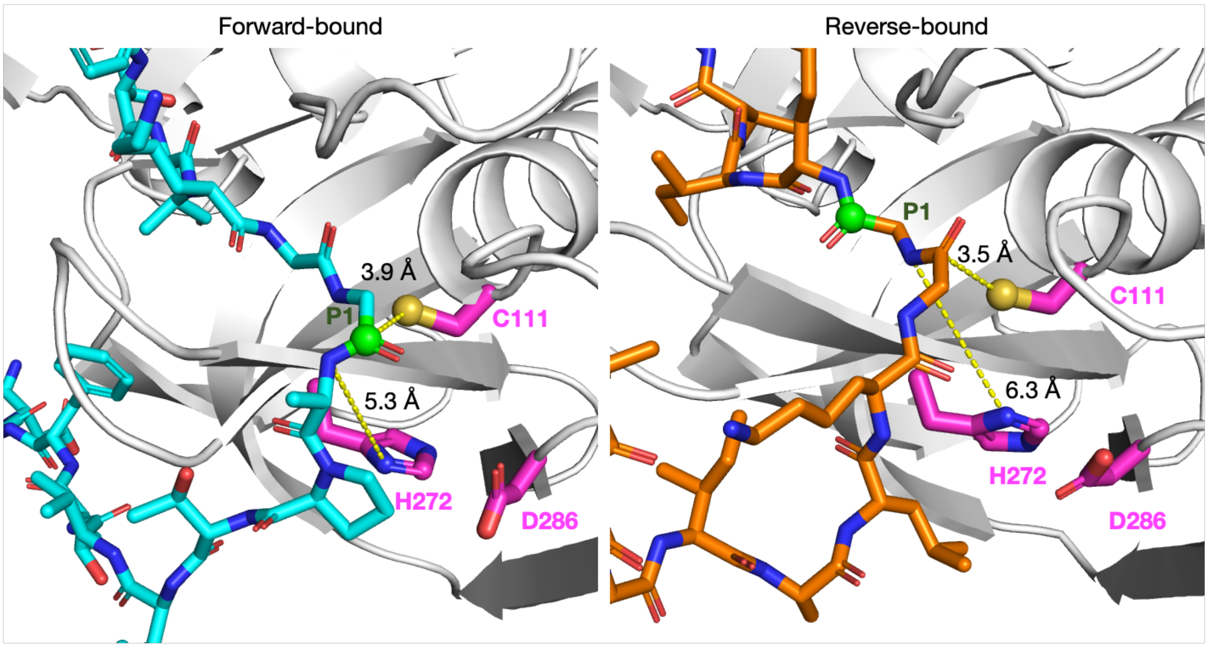

Figure S31: Comparison of the modeled forward and reverse oligopeptide binding modes near the active site, using the structures of PL^{pro} complexed with forward-bound peptide **2** (cyan) and reverse-bound peptide **3** (orange) prior to MD for illustration. The catalytic triad residue sidechains are shown as magenta sticks and are labeled. The original P1 residue backbone carbonyl carbon is shown as a green sphere. Note that in the reverse binding mode, while Cys111 is positioned to attack the P2 carbonyl carbon, in the subsequent step the substrate amide nitrogen atom would be too far from His272 to be protonated and act as the leaving group. Relevant calculated distances (yellow dashed lines) are given.

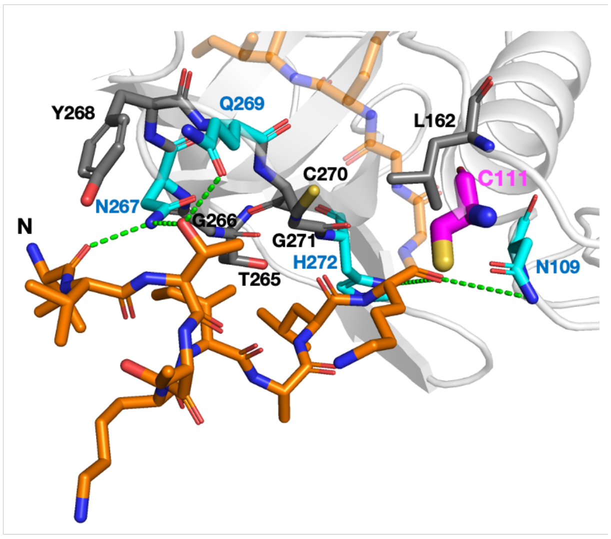

Figure S32: View of clustering-derived representative structure of Z-state PL^{pro} (white cartoon) complexed with reverse-bound peptide **3** (orange), with focus on the interactions in the S′ subsites. PL^{pro} residues involved in HBs (green dotted lines; see **Table S3**; not all significant HBs are present in these snapshots) are shown as cyan sticks. Other residues in close contact with the peptide N-terminal residues (within 4 Å) are shown in grey. The active site Cys111 is shown as magenta sticks. Clustering was performed using a 3 Å RMSD cut-off on the peptide non-hydrogen atoms using gmx cluster (gromos algorithm).[5,6] Out of 601 frames extracted every ns from the combined 3 × 200 ns MD, the number of (multimembered) clusters obtained is 29 (21).

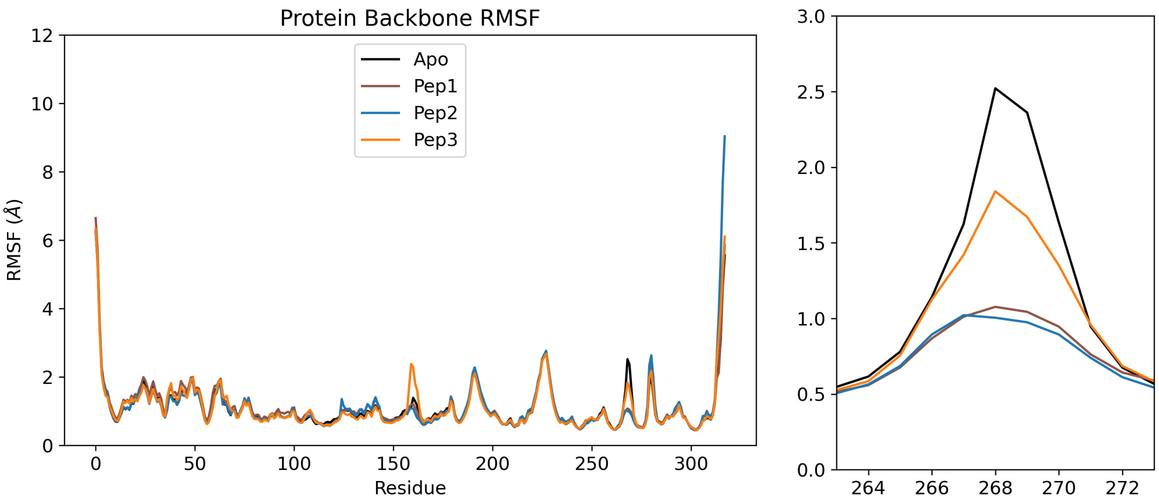

Figure S33: Per-residue RMSF of PL^{pro} backbone atoms in the combined 3 × 200 ns MD (fitted based on PL^{pro} backbone) for PL^{pro} (Z state) when apo and when complexed with each of the three forward-bound nsp peptides, (left) in full and (right) focusing around the Tyr268-containing loop region.

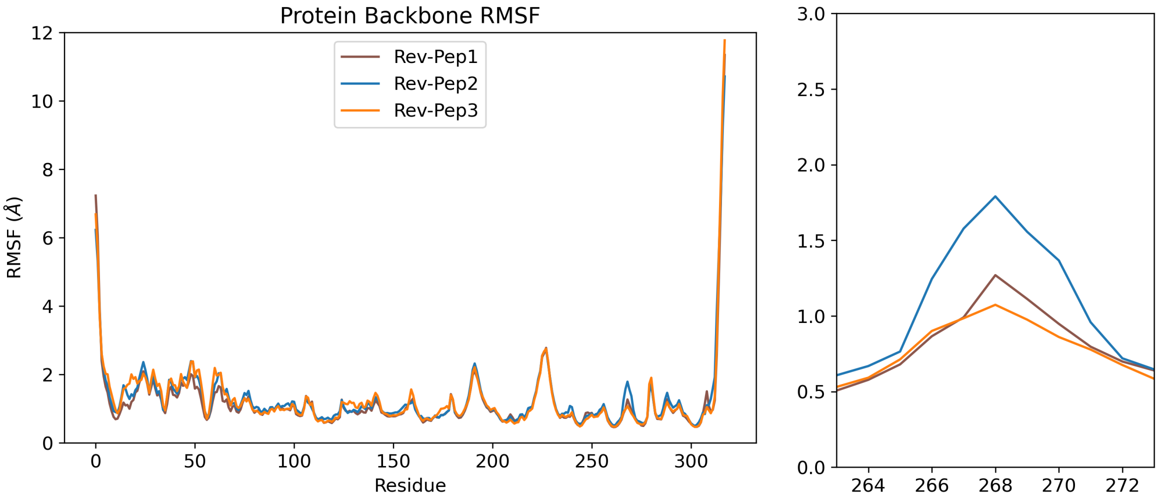

Figure S34: Per-residue RMSF of PL^{pro} backbone atoms in the combined 3 × 200 ns MD (fitted based on PL^{pro} backbone) for PL^{pro} (Z state) when complexed with each of the three reverse-bound nsp peptides, (left) in full and (right) focusing around the Tyr268-containing loop region.

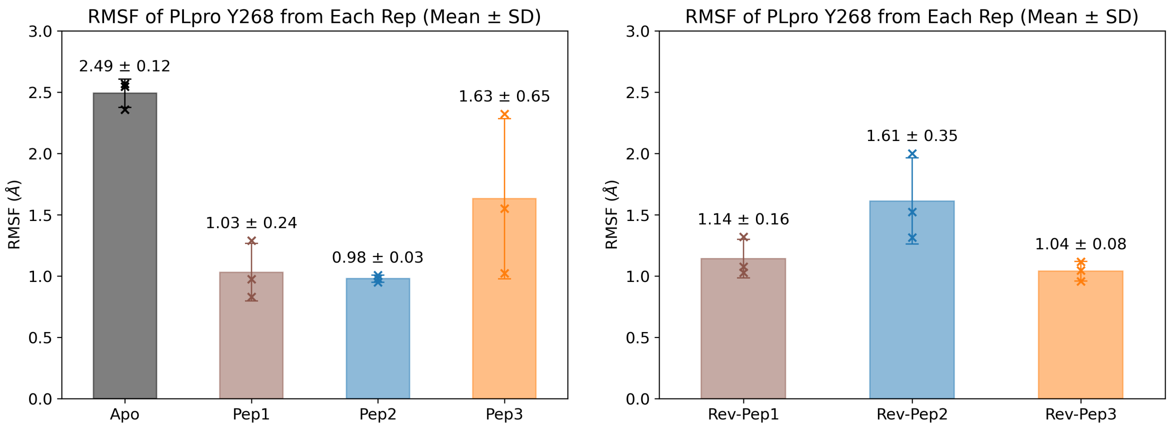

Figure S35: Per-residue RMSF of PL^{pro} Tyr268 backbone atoms in each replica of 200 ns MD (fitted based on PL^{pro} backbone) for PL^{pro} (Z state) (left) when apo and when complexed with forward-bound peptides, and (right) when complexed with reverse-bound peptides. The bar height is the arithmetic mean RMSF, and the error bar corresponds to the standard deviation (SD) across the three replicas.

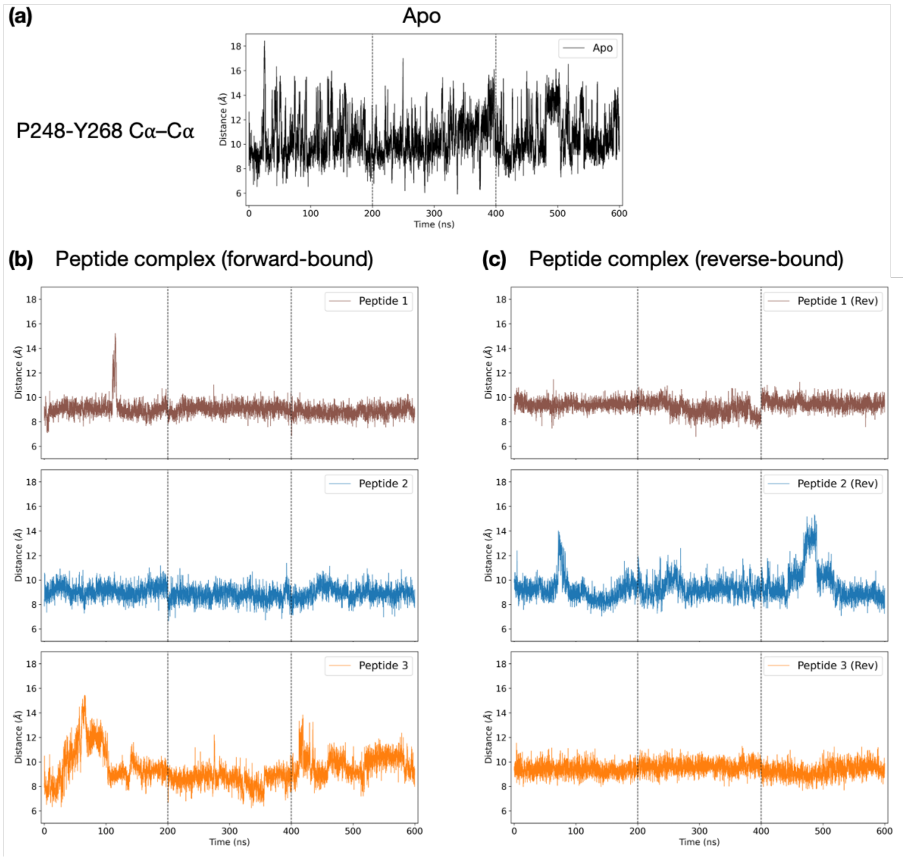

Figure S36: The evolution of the Pro248–Tyr268 Cα–Cα distance over combined 3 × 200 ns MD for PL^{pro} (a) in the apo state, and when complexed with the nsp peptides in the (b) forward and (c) reverse modes.

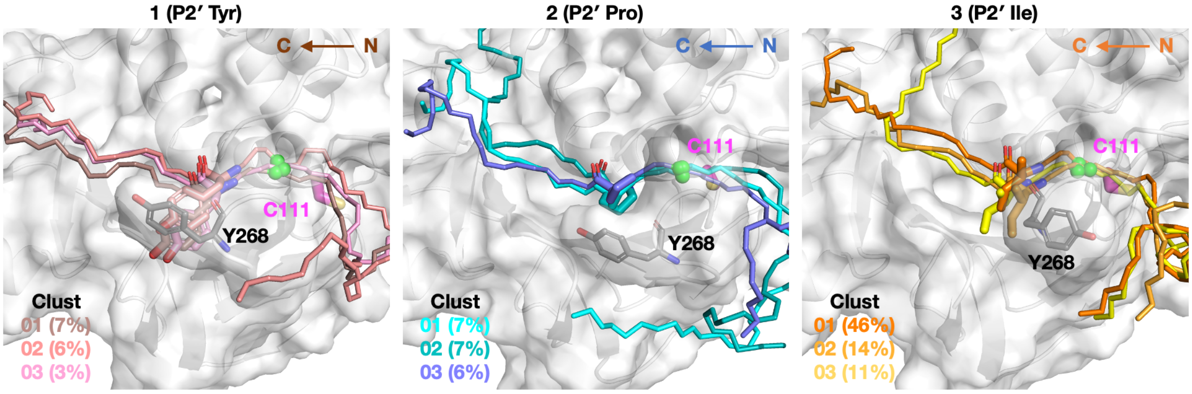

Figure S37: View of representative structures of the three most populated clusters of Z-state PL^{pro} (white cartoon and surface) complexed with reverse-bound peptides **1**, **2**, and **3**, with a focus on the S4-occupying residue (which is originally the P2′ residue). For simplification, for the rest of the peptides only the backbone atoms are shown in a uniform color. Tyr268 is shown as grey sticks, Cys111 as magenta sticks, and the original P1-Gly amide carbonyl carbon atoms as green spheres. Clustering was performed using a 3 Å RMSD cut-off on the peptide non-hydrogen atoms using gmx cluster (gromos algorithm).[5,6] Out of 601 frames extracted every ns from the combined 3 × 200 ns MD, the number of (multimembered) clusters obtained for peptides **1**, **2**, and **3** are, respectively, 226 (83), 213 (68), and 29 (21).

Table S4: Deviations of P4-P1 Cα atom positions (compared to the VIR251 in PDB 6WX4)[3] and RMSD values for the 100 highest ranked solutions from ADCP[1] docking of peptides **8**, **9**, and **10**, derived from the human proteins IRF3, PROS1, and ULK1, respectively. Solutions that pass the filter are in green. Other solutions which have Gly-Gly in S2-S1 are in orange.

Peptide 8	IRF3						*Peptide 9*	PROS1						*Peptide 10*	ULK1				
Ranking	Deviation (Å)				RMSD (Å)		Ranking	Deviation (Å)				RMSD (Å)		Ranking	Deviation (Å)				RMSD (Å)
	P4	*P3*	*P2*	*P1*				*P4*	*P3*	*P2*	*P1*				*P4*	*P3*	*P2*	*P1*	
1	20.691	14.988	11.329	12.316	15.271		1	21.076	19.259	17.103	16.880	18.658		1	12.663	7.514	3.548	4.217	7.861
2	26.166	27.233	23.456	21.644	24.723		2	18.251	12.861	12.110	10.890	13.818		2	26.802	29.800	29.013	26.499	28.064
3	30.575	32.051	27.490	22.512	28.393		3	11.785	7.754	1.270	6.462	7.784		3	30.919	31.341	28.685	25.911	29.294
4	9.673	6.512	6.460	7.313	7.602		4	31.728	28.276	23.500	19.529	26.172		4	11.646	7.326	0.722	6.814	7.685
5	17.590	12.066	9.581	5.071	11.964		5	30.648	29.071	23.579	20.387	26.249		5	25.403	27.730	22.492	22.203	24.562
6	20.291	16.738	14.997	15.492	17.006		6	0.981	0.741	0.702	0.694	0.788		6	12.550	8.660	3.674	4.020	8.096
7	30.030	31.418	27.298	22.797	28.079		7	18.226	17.470	17.154	17.163	17.509		7	45.458	41.300	38.057	33.415	39.803
8	11.887	11.666	13.353	12.782	12.441		8	34.235	30.474	30.091	28.322	30.855		8	11.376	6.749	0.269	7.174	7.525
9	10.673	10.953	9.500	11.421	10.661		9	17.485	17.538	20.884	19.271	18.847		9	6.453	0.641	0.536	0.555	3.265
10	21.082	18.648	17.556	17.096	18.659		10	28.704	27.161	24.419	23.441	26.017		10	20.371	17.614	14.836	11.094	16.343
11	19.702	19.001	14.174	15.563	17.265		11	12.565	8.398	2.960	4.667	8.046		11	11.715	6.787	0.492	6.859	7.592
12	11.265	7.065	0.615	6.908	7.499		12	33.369	31.689	29.804	26.067	30.354		12	12.975	8.051	3.630	4.065	8.107
13	12.320	11.779	12.456	12.425	12.248		13	14.189	15.573	11.775	10.268	13.114		13	27.052	26.956	24.305	20.514	24.850
14	18.997	17.115	15.134	10.545	15.764		14	17.633	15.702	13.248	9.508	14.347		14	21.116	18.839	13.351	12.353	16.820
15	17.135	17.100	15.180	10.824	15.277		15	21.699	15.364	17.451	17.363	18.117		15	31.001	26.026	24.437	19.253	25.526
16	19.973	16.753	15.648	16.511	17.299		16	37.978	32.768	31.810	27.811	32.792		16	30.274	25.702	24.355	19.348	25.222
17	13.107	11.471	10.480	12.410	11.908		17	15.371	12.243	14.213	10.322	13.179		17	20.039	19.637	14.544	10.271	16.615
18	22.687	19.700	15.029	10.059	17.535		18	21.918	19.298	16.352	13.399	18.026		18	5.990	0.314	0.204	0.326	3.006
19	20.821	18.225	16.819	16.613	18.197		19	29.433	29.209	23.557	22.084	26.278		19	31.486	25.165	24.560	19.813	25.595
20	17.576	11.120	9.732	5.296	11.783		20	40.358	35.592	33.287	26.988	34.395		20	19.005	18.689	15.009	16.053	17.273
21	12.096	11.960	12.863	12.971	12.481		21	1.135	0.429	0.547	0.825	0.783		21	14.141	13.780	13.020	13.568	13.633
22	19.244	15.705	10.521	12.630	14.893		22	14.277	9.035	3.562	4.178	8.883		22	12.411	7.737	3.733	4.061	7.815
23	17.168	15.298	17.201	15.271	16.262		23	39.317	34.383	32.064	28.067	33.705		23	6.370	0.480	0.407	0.334	3.205
24	14.089	13.870	12.312	14.472	13.710		24	30.028	23.453	20.499	12.864	22.569		24	13.952	8.293	3.821	3.846	8.556
25	19.352	17.359	14.816	12.363	16.188		25	12.449	8.372	3.093	4.471	7.979		25	20.832	18.536	14.731	11.662	16.812
26	12.480	11.524	11.101	13.938	12.309		26	21.488	18.817	14.730	9.978	16.825		26	30.233	27.101	24.454	19.728	25.669
27	17.630	11.413	9.271	5.143	11.763		27	22.502	20.023	16.919	15.477	18.928		27	22.096	19.487	14.444	15.792	18.207
28	20.641	22.132	26.152	28.800	24.644		28	26.184	25.970	29.012	29.035	27.590		28	13.144	15.459	15.164	15.139	14.755
29	25.775	25.880	30.175	32.643	28.767		29	30.986	26.733	23.596	20.537	25.755		29	38.179	32.881	30.668	25.908	32.213
30	20.205	16.639	14.992	15.520	16.961		30	31.806	28.913	25.012	19.249	26.663		30	30.727	29.031	30.082	30.484	30.088
31	24.233	22.299	26.627	28.817	25.612		31	21.046	17.326	12.489	8.450	15.576		31	32.089	27.556	27.318	22.498	27.575
32	23.044	18.123	16.737	12.799	18.052		32	19.615	14.246	12.180	9.108	14.309		32	24.393	19.035	18.574	13.644	19.291
33	19.841	18.325	14.756	15.791	17.296		33	19.701	14.034	13.578	8.600	14.521		33	20.053	16.283	15.602	10.838	16.033
34	29.485	27.341	20.815	19.940	24.737		34	20.643	18.835	16.820	16.580	18.294		34	31.005	29.692	30.207	30.504	30.356
35	22.886	19.103	18.205	18.458	19.753		35	17.205	14.089	13.719	10.832	14.142		35	38.870	34.856	33.079	27.674	33.859
36	26.523	27.270	24.076	21.748	24.999		36	13.374	8.961	3.363	4.314	8.501		36	14.647	9.749	11.869	13.805	12.659
37	26.606	22.046	17.865	14.534	20.762		37	22.831	16.179	15.177	9.894	16.668		37	19.449	18.656	13.305	9.808	15.808
38	7.044	6.165	9.760	10.641	8.604		38	17.916	15.767	11.780	8.513	13.972		38	13.992	13.712	15.522	16.683	15.025
39	23.949	20.824	17.191	15.842	19.708		39	14.400	15.122	15.782	15.866	15.304		39	38.435	36.025	32.879	26.802	33.817
40	14.874	12.558	13.756	16.505	14.496		40	16.689	17.024	17.090	17.777	17.150		40	40.739	41.688	39.097	34.607	39.127
41	19.069	18.892	14.883	11.338	16.360		41	19.004	16.033	14.334	10.930	15.355		41	15.809	10.564	11.097	9.719	12.032
42	12.268	6.742	0.644	6.856	7.800		42	40.782	35.525	32.894	28.495	34.710		42	39.401	35.295	30.683	26.048	33.234
43	13.340	16.318	17.258	21.746	17.428		43	22.383	20.613	16.273	13.634	18.551		43	28.203	25.487	24.805	25.407	26.009
44	29.522	26.607	20.674	20.107	24.552		44	17.027	12.239	9.223	7.938	12.122		44	5.488	0.778	0.376	0.405	2.785
45	11.696	11.449	13.140	12.376	12.183		45	17.695	13.742	11.542	11.610	13.874		45	30.508	29.834	29.577	29.551	29.870
46	30.424	27.337	24.964	17.480	25.503		46	26.188	21.323	22.136	21.173	22.797		46	30.065	24.869	23.670	18.878	24.693
47	18.992	19.500	15.180	16.004	17.518		47	16.113	12.168	14.415	10.869	13.543		47	12.991	17.066	22.337	28.731	21.121
48	19.040	17.955	14.451	12.287	16.161		48	27.159	26.221	24.256	20.438	24.654		48	20.242	21.853	23.716	24.397	22.610
49	12.246	6.726	0.692	6.839	7.785		49	30.480	30.550	25.052	24.015	27.689		49	21.073	18.474	15.922	11.910	17.181
50	21.462	17.141	13.699	13.579	16.782		50	14.814	12.838	14.369	12.699	13.711		50	17.967	13.439	11.692	7.416	13.182
51	27.908	28.925	25.734	24.096	26.732		51	27.023	26.952	24.537	20.713	24.939		51	37.171	32.765	31.211	26.662	32.172
52	25.663	22.495	24.807	24.688	24.441		52	39.351	32.574	30.144	27.115	32.609		52	12.889	8.350	3.605	4.194	8.161
53	29.671	27.910	23.621	18.261	25.252		53	8.913	2.015	7.871	13.392	9.011		53	18.799	16.341	20.513	20.978	19.244
54	18.734	17.404	20.405	21.843	19.668		54	27.557	26.375	24.011	19.885	24.632		54	35.488	28.976	24.772	20.153	27.923
55	18.370	12.512	9.910	5.155	12.438		55	26.560	26.679	29.708	30.054	28.298		55	13.577	12.056	11.402	13.414	12.645
56	27.075	26.458	23.556	19.451	24.322		56	19.402	13.226	14.128	9.822	14.555		56	27.469	29.756	26.799	25.072	27.326
57	21.372	18.220	13.268	14.190	17.074		57	37.838	32.868	33.611	29.328	33.548		57	7.145	0.376	0.309	0.420	3.587
58	27.416	22.950	21.197	16.348	22.332		58	0.814	0.227	0.354	0.693	0.575		58	28.243	24.220	24.823	20.538	24.608
59	11.046	7.784	7.255	9.063	8.908		59	13.419	13.331	13.502	13.993	13.564		59	18.359	14.162	16.956	17.214	16.744
60	18.556	11.710	9.933	5.081	12.308		60	15.034	10.638	10.345	8.994	11.479		60	24.948	21.134	20.163	16.328	20.870
61	30.378	32.123	27.672	23.238	28.550		61	28.684	26.185	22.582	22.923	25.218		61	13.157	10.947	12.003	11.043	11.821
62	29.465	25.386	20.854	13.210	23.033		62	32.653	29.050	23.693	18.989	26.609		62	15.153	11.757	10.722	12.871	12.732
63	18.813	14.783	14.520	15.039	15.886		63	21.482	18.976	17.415	17.097	18.822		63	30.944	30.191	27.836	29.789	29.712
64	19.235	17.356	20.577	20.606	19.489		64	21.759	17.088	13.132	13.918	16.820		64	31.708	27.685	23.867	16.933	25.633
65	16.665	11.720	11.730	8.710	12.535		65	22.324	22.765	18.469	14.002	19.709		65	12.798	13.036	11.485	9.454	11.779
66	28.216	26.191	20.952	18.130	23.716		66	42.391	38.717	37.043	33.314	38.007		66	33.496	35.322	33.082	30.927	33.243
67	10.723	7.143	7.167	7.800	8.340		67	18.926	21.189	15.219	12.485	17.282		67	44.035	43.143	39.384	34.631	40.469
68	13.189	7.837	8.228	9.332	9.876		68	12.576	14.023	15.133	14.911	14.196		68	6.268	0.348	0.677	1.028	3.199
69	29.453	27.523	29.247	28.515	28.695		69	32.491	29.851	25.878	19.487	27.369		69	45.462	41.439	37.403	33.058	39.610
70	21.829	21.695	23.691	26.053	23.384		70	32.214	27.082	26.563	22.656	27.341		70	24.552	27.003	22.592	21.379	23.976
71	12.551	12.164	13.986	12.562	12.835		71	11.665	8.976	12.068	12.049	11.263		71	26.778	25.074	24.809	21.665	24.651
72	19.477	16.851	20.195	21.865	19.680		72	21.107	19.615	18.674	13.991	18.539		72	5.391	0.830	0.368	0.686	2.755
73	17.329	12.440	10.789	10.510	13.056		73	30.662	28.185	29.179	27.507	28.907		73	30.960	29.574	30.164	30.027	30.185
74	16.904	16.583	15.971	16.218	16.423		74	31.275	29.465	26.133	20.521	27.159		74	30.289	29.590	29.719	28.374	29.501
75	34.709	36.127	34.818	33.596	34.824		75	21.169	19.693	24.833	22.877	22.226		75	12.836	11.994	14.555	14.711	13.572
76	33.779	36.609	35.487	30.978	34.279		76	31.331	28.389	23.830	19.075	26.074		76	28.680	28.638	22.228	19.308	25.048
77	15.351	11.047	10.719	10.344	12.037		77	21.110	14.497	14.503	10.332	15.596		77	11.266	6.657	0.383	7.314	7.498
78	11.793	10.642	10.062	13.661	11.621		78	30.036	25.670	21.575	21.574	24.960		78	25.757	21.168	18.450	14.354	20.359
79	26.638	28.374	22.533	21.123	24.842		79	36.648	35.685	33.309	28.223	33.625		79	12.338	8.272	3.351	4.398	7.925
80	10.620	10.878	10.094	11.854	10.880		80	13.411	8.909	3.122	4.651	8.524		80	20.202	16.316	15.598	11.222	16.152
81	13.312	13.470	10.736	14.199	12.995		81	28.468	26.260	23.207	22.892	25.311		81	30.268	30.852	29.007	24.920	28.855

82	34.840	34.727	30.609	29.112	32.420		82	38.657	33.586	33.706	29.521	34.022		82	63.529	57.479	53.332	47.791	55.830
83	23.462	21.572	15.908	12.209	18.828		83	27.877	26.274	24.054	19.675	24.663		83	31.400	26.767	26.052	21.216	26.605
84	19.064	14.861	14.321	14.746	15.865		84	16.768	17.975	17.085	17.719	17.393		84	4.044	3.996	10.493	16.966	10.372
85	28.433	27.112	20.999	19.472	24.308		85	14.685	12.910	14.588	12.465	13.698		85	14.347	11.485	11.522	12.470	12.510
86	11.606	7.171	0.733	6.645	7.597		86	30.389	26.999	25.251	22.592	26.459		86	5.613	0.951	0.891	0.924	2.918
87	15.770	10.738	10.010	7.412	11.392		87	18.461	15.648	13.876	15.514	15.960		87	22.803	20.597	14.633	12.274	18.090
88	12.523	13.524	11.707	15.679	13.441		88	14.463	16.910	13.376	13.006	14.519		88	14.643	12.631	12.550	10.327	12.630
89	45.239	45.557	48.074	46.112	46.259		89	38.390	32.932	31.981	27.958	33.026		89	24.102	19.276	18.604	14.628	19.446
90	22.863	18.192	16.886	12.580	18.007		90	23.658	22.885	21.198	19.149	21.791		90	33.137	27.723	25.252	21.145	27.163
91	20.126	16.732	17.682	14.132	17.301		91	36.404	30.811	28.744	24.192	30.356		91	22.105	23.275	21.697	19.275	21.637
92	24.487	25.176	27.819	30.362	27.061		92	12.172	7.650	1.364	6.525	7.923		92	11.144	6.626	0.681	7.370	7.465
93	17.701	11.093	9.375	5.372	11.760		93	18.423	13.003	12.023	10.899	13.891		93	13.714	12.225	12.306	8.841	11.907
94	30.556	29.966	32.072	32.570	31.309		94	29.586	29.605	31.566	31.064	30.468		94	11.985	11.423	11.155	10.385	11.252
95	20.159	20.271	15.490	16.705	18.278		95	24.271	24.022	23.634	24.265	24.049		95	16.431	14.010	17.500	18.366	16.657
96	17.734	15.158	17.133	15.121	16.328		96	17.338	10.679	9.691	8.998	12.140		96	11.805	7.187	0.603	6.736	7.693
97	27.092	24.839	22.227	20.378	23.771		97	1.332	0.508	0.178	0.382	0.743		97	19.636	12.804	11.247	7.828	13.577
98	19.454	20.097	18.269	19.247	19.278		98	27.880	22.269	17.417	10.810	20.576		98	32.158	30.987	31.349	31.800	31.576
99	32.660	31.484	32.540	31.449	32.038		99	34.652	32.575	31.146	27.991	31.684		99	23.225	24.207	22.031	20.372	22.504
100	12.424	12.057	12.723	10.933	12.054		100	40.157	33.291	28.952	26.947	32.731		100	12.447	7.368	3.541	4.324	7.753

Table S5: Deviations of P4-P1 Cα atom positions (compared to the VIR251 in PDB 6WX4)[3] and RMSD values for the 100 highest ranked solutions from ADCP[1] docking of peptide **11** derived from the human protein ATG7. None of the solutions passed the filter or had a Gly-Gly in S2-S1. Solutions where the peptide passes through S2-S1 are in yellow.

Peptide 11	ATG7				
**Ranking**	**Deviation(Å)**				**RMSD(Å)**
	P4	*P3*	*P2*	*P1*	
1	24.146	18.466	14.408	8.272	17.321
2	22.661	23.382	21.177	15.339	20.880
3	27.039	30.261	27.297	26.712	27.863
4	21.213	21.798	20.288	15.273	19.812
5	28.858	26.516	23.809	24.188	25.922
6	25.972	25.956	25.364	22.815	25.060
7	26.087	26.573	23.289	21.794	24.515
8	29.679	31.882	29.182	27.231	29.540
9	29.074	24.228	18.254	12.004	21.850
10	26.997	21.082	21.437	18.325	22.185
11	43.782	37.931	34.472	27.697	36.439
12	27.916	28.063	25.421	21.954	25.957
13	23.682	23.583	21.601	15.514	21.356
14	34.681	34.114	32.082	30.547	32.897
15	23.121	21.828	19.609	12.732	19.734
16	13.513	7.986	10.109	9.339	10.438
17	39.861	38.628	40.778	41.826	40.291
18	20.335	17.590	11.596	6.524	15.000
19	26.799	22.570	23.739	18.063	23.008
20	9.246	3.158	3.166	7.696	6.417
21	17.448	22.418	26.469	29.720	24.448
22	24.168	24.618	25.501	23.925	24.560
23	23.340	17.549	13.661	8.147	16.626
24	29.742	32.834	30.845	26.788	30.132
25	16.359	11.543	9.929	8.919	12.031
26	21.760	20.369	14.298	9.299	17.170
27	25.744	25.200	21.251	19.254	23.022
28	35.985	34.227	31.426	25.205	31.974
29	25.468	20.450	17.549	11.722	19.444
30	18.149	17.654	15.596	11.627	15.965
31	23.469	22.271	21.732	15.577	20.987
32	32.757	28.374	23.792	22.195	27.096
33	12.383	12.076	9.240	7.307	10.464
34	23.294	21.501	18.033	14.224	19.573
35	26.815	22.678	20.954	15.843	21.929
36	21.106	20.956	15.086	13.041	17.904
37	17.799	13.363	7.549	4.815	11.995
38	11.963	8.214	12.195	15.410	12.214
39	24.486	24.894	25.494	22.736	24.424
40	35.218	35.235	33.692	27.884	33.145
41	16.111	16.971	12.972	11.562	14.573
42	19.689	13.857	10.849	9.782	14.080
43	25.753	20.952	15.913	14.413	19.769
44	29.451	28.255	23.957	21.827	26.058
45	27.441	24.773	25.094	22.394	24.990
46	36.855	35.511	33.371	34.126	34.991
47	23.834	24.400	24.154	22.173	23.656
48	8.088	4.003	4.028	7.231	6.123
49	32.119	27.936	25.673	20.879	26.959
50	34.957	30.435	27.744	21.984	29.161
51	27.168	30.062	27.429	25.690	27.632
52	16.750	15.689	10.854	8.123	13.328
53	16.082	16.522	17.695	19.788	17.580
54	29.480	28.185	22.300	20.323	25.366
55	27.406	21.161	20.718	17.509	21.992
56	20.743	18.213	14.725	13.384	17.014
57	28.452	25.228	23.794	22.963	25.196
58	44.517	38.651	34.920	29.808	37.362
59	43.926	38.541	35.424	28.655	37.050
60	28.481	31.029	26.524	26.383	28.167
61	28.568	25.866	24.765	19.437	24.881
62	26.914	20.908	21.625	17.729	22.042
63	26.867	27.681	27.292	30.578	28.142
64	30.946	33.258	29.226	27.193	30.238
65	44.062	37.789	36.976	32.794	38.119
66	20.063	14.382	11.671	10.054	14.549
67	35.626	32.205	28.949	23.406	30.382
68	3.947	8.819	13.124	14.870	11.031
69	15.958	15.415	10.769	7.879	12.945
70	22.895	16.141	12.849	8.445	15.978
71	25.323	19.264	18.567	12.420	19.438
72	17.197	22.859	26.480	29.292	24.380
73	32.720	30.270	24.796	22.179	27.810
74	28.921	26.225	23.383	23.665	25.646
75	19.772	18.516	19.601	15.553	18.438
76	31.739	25.947	22.864	18.456	25.219
77	17.466	22.216	27.582	32.940	25.712
78	18.950	19.698	14.874	13.083	16.878
79	44.167	38.874	35.751	30.657	37.683
80	31.887	29.713	26.523	25.003	28.409
81	26.758	30.275	27.049	26.545	27.699

82	33.434	30.317	25.931	18.718	27.657
83	21.738	20.458	21.000	17.991	20.345
84	27.989	27.519	22.246	20.844	24.849
85	26.843	22.182	19.922	14.634	21.352
86	23.842	21.479	18.132	13.534	19.632
87	24.162	22.162	16.957	13.548	19.660
88	19.540	13.959	11.139	9.690	14.095
89	18.141	14.707	10.166	5.392	13.018
90	17.780	22.471	25.685	28.218	23.859
91	27.179	25.918	26.832	24.900	26.222
92	23.165	17.632	13.196	9.198	16.630
93	19.612	16.053	11.628	6.053	14.267
94	16.920	22.975	26.466	28.589	24.145
95	3.878	4.159	8.390	12.619	8.093
96	11.413	12.372	14.710	11.489	12.567
97	22.907	17.155	13.205	8.290	16.295
98	25.420	26.473	23.376	21.716	24.316
99	27.590	27.642	24.082	23.276	25.725
100	27.255	23.527	23.373	17.906	23.256

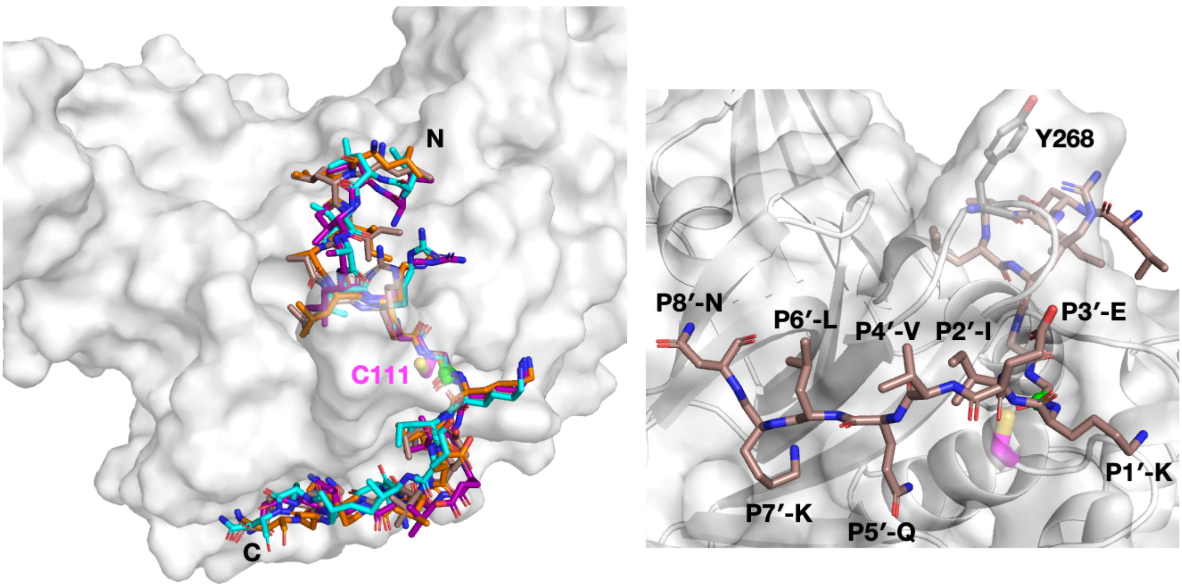

Figure S38: Docked conformations of PROS1-derived peptide **9** that passed the applied filter, *i.e.*, the four Cα atoms of P4-P1 have to be within 2 Å of the corresponding Cα atoms of VIR251[3]. The poses include ranked solutions 06 (brown), 21 (cyan), 58 (orange), and 97 (purple), with the general positioning of the terminals labeled. The P1 amide carbonyl carbon is shown as a green sphere. A magnified view of the P′ residues in solution 06 is shown, along with Tyr268 (grey sticks) in the BL2 loop.

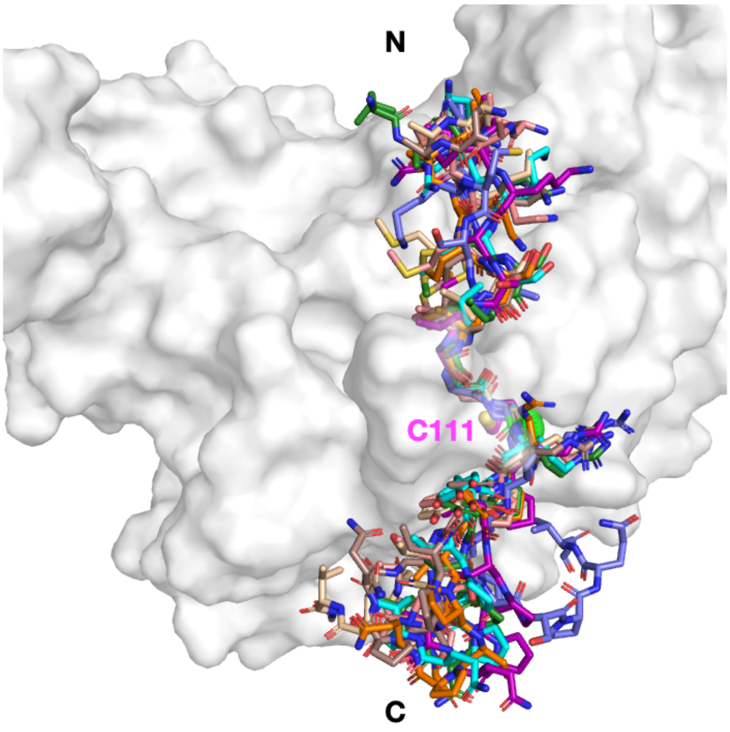

Figure S39: Docked conformations of ULK1-derived peptide **10** where P3-P1 Cα deviations were within 2 Å, but not P4. The poses include ranked solutions 09 (brown), 18 (cyan), 23 (orange), 44 (purple), 57 (dark green), 68 (slate), 72 (wheat), and 86 (salmon), with the general positioning of the terminals labeled. The P1 amide carbonyl carbon is shown as a green sphere.

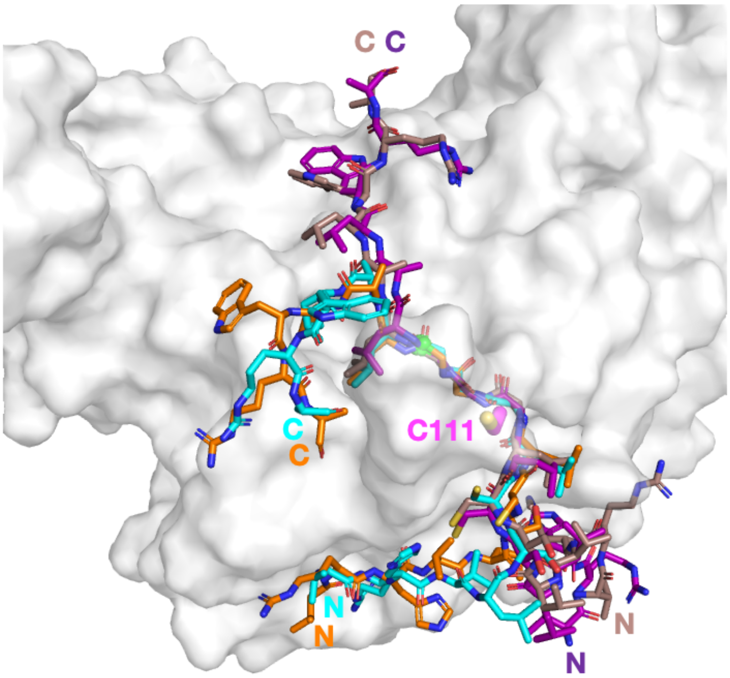

Figure S40: Docked conformations of IRF3-derived peptide **8** that are bound in reverse with the P2-Gly in S2 and P3-Gly in S1. The poses include ranked solutions 12 (brown), 42 (cyan), 49 (orange), and 86 (purple), with individual N- and C-terminals labeled. The P1 amide carbonyl carbon is shown as a green sphere.

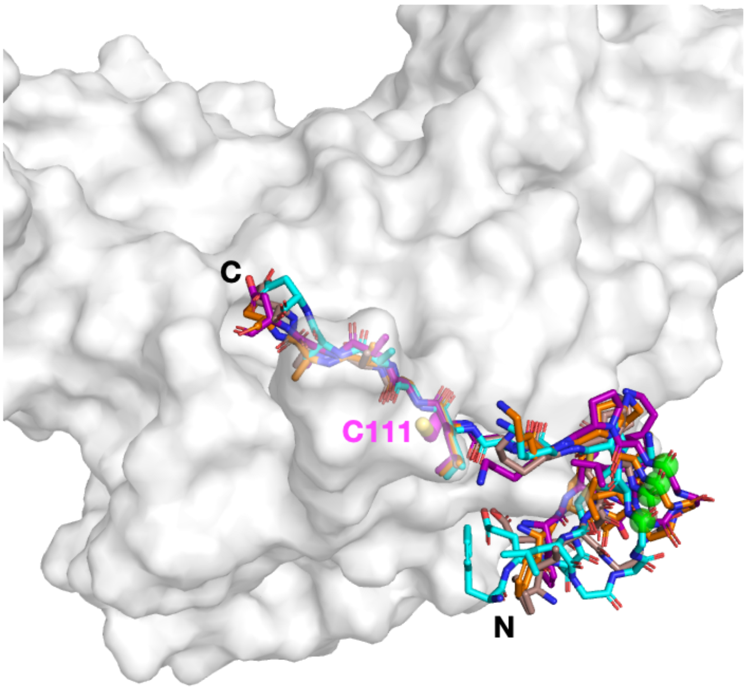

Figure S41: Docked conformations of ATG7-derived peptide **11** that are bound in reverse with the P5′-P8′ LAAA residues in the S1-S4 subsites, respectively. The poses include ranked solutions 02 (brown), 4 (cyan), 13 (orange), and 31 (purple), with the general positioning of the terminals labeled. The P1 amide carbonyl carbon is shown as a green sphere.

Table S6: Assignment of PL^{pro} histidine protonation states from the structure of PDB 6WX4.[3] Neutral N^{δ}-protonated, neutral N^{ε}-protonated, and doubly protonated histidines are named HID, HIE, and HIP respectively.

HIS	Assignment	Reason
17	HIP	HE2 hydrogen bond (HB) donor to the Glu67 carboxylate (N-O 2.8 Å). Solvent exposed on ND1 side. pK_a calculations (H++ and PROPKA)[9,10] consistently suggest double protonation at pH 7, with negatively charged residues Asp12 and Glu67 nearby, but no positively charged residues nearby.
47	HIE	ND1 HB acceptor for Ser49 sidechain OH (N-O 3.0 Å) or Ser49 backbone NH (N-N 3.1 Å).
50	HIE	ND1 HB acceptor for Tyr27 sidechain OH (N-O 2.9 Å) or His47 backbone NH (N-N 3.2 Å).
73	HIE	No clear HB partners nearby, assuming HIE.
89	HID	NE2 solvent exposed. ND1/HD1 HB donors to Ser85 backbone O (N-O 3.4 Å).
175	HID	ND1/HD1 HB donors to Tyr171 backbone O (N-O 3.0 Å).
255	HIE	NE2/HE2 HB donors to Lys279 backbone O (N-O 2.6 Å).
272	HIE/HIP	Part of the catalytic triad. HE2 HB donor to Asp286 (N-O 3.0 Å). ND1 side solvent exposed; protonation state likely depends on the charge state of Cys111.<br>Subsequent QM/MM-US calculations (**Figures S7-S11**) suggested HIP is favored.
275	HID	NE2 HB acceptor for Gln122 NE2 (N-N 2.8 Å), which in turn is a HB donor to backbone O of Leu118 (N-O 3.0 Å) and HB acceptor, via its OE1, for T277 sidechain OH (O-O 2.6 Å).

References

1. Y. Zhang and M. F. Sanner, *Bioinformatics*, 2019, **35**, 5121-5127.
2. L. Brewitz, J. Kamps, P. Lukacik, C. Strain-Damerell, Y. Zhao, A. Tumber, T. R. Malla, A. M. Orville, M. A. Walsh and C. Schofield, *ChemMedChem*, 2022, **17**, e202200016.
3. W. Rut, Z. Lv, M. Zmudzinski, S. Patchett, D. Nayak, J. Snipas Scott, F. El Oualid, T. Huang Tony, M. Bekes, M. Drag and K. Olsen Shaun, *Sci. Adv.*, 2020, **6**, eabd4596.
4. A. Grossfield, WHAM: an implementation of the weighted histogram analysis method, http://membrane.urmc.rochester.edu/content/wham/, (accessed 2020-04-17).
5. M. J. Abraham, T. Murtola, R. Schulz, S. Páll, J. C. Smith, B. Hess and E. Lindahl, *SoftwareX*, 2015, **1-2**, 19-25.
6. X. Daura, K. Gademann, B. Jaun, D. Seebach, W. F. van Gunsteren and A. E. Mark, *Angew. Chem. Int. Ed.*, 1999, **38**, 236-240.
7. W. G. Touw, C. Baakman, J. Black, T. A. H. te Beek, E. Krieger, R. P. Joosten and G. Vriend, *Nucleic Acids Res.*, 2015, **43**, D364-D368.
8. W. Kabsch and C. Sander, *Biopolymers*, 1983, **22**, 2577-2637.
9. R. Anandakrishnan, B. Aguilar and A. V. Onufriev, *Nucleic Acids Res.*, 2012, **40**, W537-W541.
10. M. H. M. Olsson, C. R. Søndergaard, M. Rostkowski and J. H. Jensen, *J. Chem. Theory Comput.*, 2011, **7**, 525-537.
